# Inoculation of apple plantlets with *Rhodococcus pseudokoreensis* R79^T^ enhances diversity and modulates the structure of bacterial rhizosphere communities in soil affected by apple replant disease

**DOI:** 10.1186/s12870-025-06747-9

**Published:** 2025-05-28

**Authors:** Sarah Benning, Fatma M. Mahmoud, Pamela Espindola-Hernandez, Benye Liu, Karin Pritsch, Viviane Radl, Jana Barbro Winkler, Traud Winkelmann, Ludger Beerhues, Michael Schloter

**Affiliations:** 1 Research Unit for Comparative Microbiome Analysis, Helmholtz Munich, German Research Center for Environmental Health, Neuherberg, Germany; 2 Botany and Microbiology Department, Faculty of Science, Suez Canal University, Ismailia, Egypt; 3 Institute of Pharmaceutical Biology, Technische Universität Braunschweig, Braunschweig, Germany; 4 Research Unit for Environmental Simulations, Helmholtz Munich, German Research Center for Environmental Health, Neuherberg, Germany; 5 Institute of Horticultural Production Systems, Leibniz University, Hannover, Germany; 6 Chair for Environmental Microbiology, TUM School of Life Sciences, Technical University, Munich, Germany; 7 Present Address: Department of Safety and Area Management, Helmholtz Munich, German Research Center for Environmental Health, Neuherberg, Germany

**Keywords:** *Rhodococcus*, Apple replant disease, Degradation of benzoate and biphenyls, Phytoalexins, Rhizosphere microbiome, Bioinoculum.

## Abstract

**Background:**

Apple replant disease (ARD) represents a dysbiotic rhizosphere condition potentially driven by root exudates including phytoalexins at the root–soil interface. A promising mitigation strategy could be the application of bioinoculants that reduce these compounds and foster a diverse microbiome. This study investigated the effects of *Rhodococcus pseudokoreensis* R79^T^, a strain with benzoate-degrading capabilities and genetic potential to degrade biphenyls, on the rhizosphere microbiome of apple plantlets grown in ARD-affected soil in a greenhouse experiment.

**Results:**

We applied R79^T^ at 10⁶ to 10⁹ CFU/ml, assessing its impact on bacterial 16S rRNA diversity and abundance, as well as the abundance of biphenyl dioxygenase (*bphd)* genes. Eight weeks post-inoculation reads of strain R79^T^ persisted in the rhizosphere, particularly at higher inoculation levels. Inoculation enhanced bacterial diversity and *bphd* gene abundance, with significant shifts in community composition. Key responders included members of Gaiellales, which increased, and *Streptomyces*, which decreased. Co-occurrence network analysis revealed that inoculation promoted positive interactions, more homogeneous connectivity, and a higher degree of connections. Effects on bacterial community structure varied significantly with inoculation concentration.

**Conclusions:**

The fact that R79^T^ enhanced rhizosphere bacterial diversity and modulated community composition in ARD-affected soil highlights the potential of R79^T^ to reshape microbial interactions. Further research is needed to elucidate the mechanisms underlying these effects, including studies on in situ degradation of phytoalexins and inoculation of R79^T^ alongside bacteria for plant growth promotion (PGP) in synthetic communities for elevated efficiency against ARD.

**Supplementary Information:**

The online version contains supplementary material available at 10.1186/s12870-025-06747-9.

## Background

Application of microbial inoculants in agriculture for improving plant and soil performance is a topic of high importance in current research for promoting sustainable, economic, and ecological measures [1, 2]. Bacterial and fungal inoculants are used to improve plant performance through direct plant growth promotion (PGP), antimicrobial properties or enhancement of nutrient uptake [3–5]. Microbial inoculants are also used to indirectly promote plant health and resilience, by modulating the rhizosphere microbiome. The enrichment of beneficial microbial taxa from the native microbial community, increase or stabilisation of overall microbial diversity and supression of pathogens have been associated with improved soil health, nutrient cycling, and plant performance [6–8]. However, more focus needs to be put on the actual analysis of these microbiome changes, to be aware of unwanted non-target effects of introduction of foreign microbes into the soil [9]. Promising results have been achieved by inoculating single strains or consortia to directly improve the growth and performance of various crops such as tomato, wheat, rice, soybean, and other agricultural and horticultural plants [3, 10, 11]. For apple replant disease (ARD), inoculation with actively plant growth promoting microbes yielded inconsistent results regarding improved plant performance [12, 13], emphasizing the need for alternative treatments to common PGP assays. ARD is a widespread problem globally affecting growth and yield in tree nurseries and apple orchards when apple trees are replanted in soil previously used for apple cultivation. Common ARD disease symptoms include blackening/browning of roots, reduction in lateral roots and root biomass, and higher prevalence for plant pathogens [14, 15]. It is a non-systemic plant reaction, affecting roots only where they are in contact with the diseased soil [16], and biotic stress response genes induced by ARD in the roots have not been found to have a similar response in aboveground tissue [17]. A high inconsistency and local heterogeneity of disease severity, microbial community composition, and differences in pathogen abundance for different geographic regions, seasons, soils, and rootstocks [18] complicate a deeper understanding of ARD.

ARD is hypothesized to be a physiologically disturbed response of the plant accompanied by alterations in the rhizosphere microbiome [15, 19], and the accumulation of aromatic compounds in soil and roots [20]. Apple roots produce and exude various aromatic compounds, including phytoalexins like biphenyls and dibenzofurans, a group of naturally occurring defense compounds produced by plants of the rosaceous subtribe Malinae in response to pathogen attack or other biotic stresses [21, 22]. Phytoalexins were shown to correlate with ARD symptoms [23–25] and have been hypothesized to contribute to the disease development [26] by not only inhibiting the growth of potential pathogens, but also of beneficial soil microorganisms [27]. In addition to phytoalexins, other aromatic compounds such as phenolic acids, benzoate, phlorizin, and vanillin were found in higher concentration in ARD soil [28], and root exudates of ARD-affected plants were enriched in flavonoids [24, 29]. Impaired growth of apple plantlets was directly related to an accumulation of phenolic compounds, especially phlorizin, in soil [20]. In addition to studies that reported altered soil microbial community composition in ARD soil and a lower microbial diversity [9, 11], a metagenome study revealed fewer genes coding for the degradation of aromatic compounds like benzoate and elevated numbers for genes involved in stress response in ARD soil compared to control soil [30].

Efforts to biologically improve the growth of apple plantlets in ARD-affected soils thus may require not only the use of classical plant growth promoting or biocontrol strains, but also the introduction of bioinoculants that enhance the overall microbial diversity again while being able to tolerate high concentrations of aromatic compounds. As a suitable candidate, we identified a *Rhodococcus* strain isolated from apple rhizosphere in non-replant soil, which formed a new species in the genus *Rhodococcus* (*R. pseudokoreensis*) [31]. The type strain *R. pseudokoreensis* R79^T^ showed high genetic potential regarding the tolerance and degradation of aromats, especially benzoates and biphenyls [32]. In addition, the strain was shown to grow with several biphenyl phytoalexins in environmental concentrations in vitro [27], as well as degrade benzoate in vitro (data not shown). Other phenotypes in the genus *Rhodococcus* revealed potential in bioremediation as well as the ability to degrade a wide range of organic pollutants, including polychlorinated biphenyls [33–35] and aromatic compounds derived from lignin depolymerization [36], to promote plant growth in the presence of toxic chromate [37], or to degrade biphenyl, while promoting lateral root growth and fresh weight of *Arabidopsis thaliana* [38].

Thus, we used *R. pseudokoreensis* R79^T^ as a novel bioinoculant based on its genetic potential to degrade biphenyls and in vitro activity on benzoate. We hypothesized that these traits may support the establishment of a more diverse and beneficial rhizosphere microbiome. While inoculation with microbial consortia was shown to provide even better results regarding PGP and pathogen oppression than single strain inoculations [39], understanding the impact of individual strains on the plant-microbiome holobiont is a crucial first step. We analysed the effects of inoculation with different concentrations of *R. pseudokoreensis* R79^T^ on the rhizosphere microbiome and excluded potential negative effects on the young apple plantlets in a short-term greenhouse experiment. Following on from a study that demonstrated a higher impact of inoculation concentration on the resident microbial community than repeated inoculations [40], we hypothesized that there would be an optimal range of concentration for inoculation, which leads to the most stable community structure and poses the least amount of stress for the plant. We analyzed rhizosphere soil samples of apple plantlets grown in ARD soil and inoculated with *R. pseudokoreensis* R79^T^ at four concentrations (2.5*10^6^ to 2.5*10^9^ CFU/ml) and sterile H_2_O. The concentrations were chosen to mimic commercially used concentrations (10^7^, 10^8^ CFU/ml) and a magnitude lower and higher, to assess possible limitations and stress reactions of the plants. Based on 16 S rRNA gene amplicons, we analyzed the impact on bacterial community diversity, composition, and co-occurrence network structure. We additionally quantified the bacterial abundance, particularly the abundance of biphenyl dioxygenase (*bphd*) genes by qPCR. Plant responses were assessed by measuring plant growth and the phytoalexin concentration in the roots. In this study, we explored the effects of root inoculation of a bacterium with the genetic potential to degrade aromatic compounds on the rhizosphere of apple plantlets growing in ARD soil, beyond PGP or biocontrol.

## Methods

### Experimental setup and conditions

A greenhouse experiment was conducted in May / June 2021 to study the effect of inoculation with *R*. *pseudokoreensis* R79^T^ in different concentrations on the growth of apple plantlets in ARD-affected soil and on the respective bacterial community composition at the plant-soil interface. Soil with proven apple history with typical ARD symptoms shown in a greenhouse trial (soil C in Orth et al. (2024) [41], ARD biotest like in Yim et al. 2015 [42]) from Schleswig-Holstein, northern Germany, was obtained from an apple tree nursery in April 2021. The soil (gleyic Podzol) was characterized as sand, with 93.7% sand, 3.7% silt, and 2.6% clay [41]. The soil was sieved and homogenized using an 8 mm sieve before the experiment and supplemented with 2 g L^-1^ Osmocote Exact 3–4 M (16% *N* + 9% P₂O₅ + 12% K₂O + 2% MgO, ICL Deutschland, Nordhorn, Germany) to ensure a continuous sufficient supply of nutrients for the plants.

As a bacterial inoculant, we used strain *R. pseudokoreensis* R79^T^ further termed ’R79^T^’ [31] isolated from apple rhizosphere and tested in previous studies [32, 43]. For inoculation, R79^T^ was precultured from glycerol stock in actinomyces broth (Sigma-Aldrich) at 28 °C, 200 rpm, for 2 days, then transferred to the same cultivation medium and grown at 28 °C until it reached an OD_600_ of ~ 0.8. The culture was transferred into a 50 mL falcon tube and centrifuged for 20 min at 3720 xg. The supernatant was discarded, and the bacterial pellet was washed twice with sterile H_2_O. Finally, the pellet was resuspended in 100 ml sterile H_2_O.

In vitro propagated and acclimatized 12 weeks old plantlets of the ARD-sensitive rootstock M26 (see [44] for propagation details) were cleaned from their substrate, shortly washed in sterile H_2_O, and then shoot length was measured. For inoculation, the roots were dipped for 5 min in 100 ml of the inoculation solution, with 2.5*10^6^ CFU/ml, 2.5*10^7^ CFU/ml, 2.5*10^8^ CFU/ml, 2.5*10^9^ CFU/ml or sterile H_2_O as control, respectively. The treatments are called IC6, IC7, IC8, IC9 (IC = inoculation concentration) and control. The apple plantlets were planted in ARD soil in 10 × 11 × 7 cm pots, 10 replicates per treatment, and watered carefully. They were grown for 8 weeks in the greenhouse, with temperatures of 22 °C during the day and 18 °C at night, natural photoperiod (29/04–24/06) and watering to about 60% of maximum soil water holding capacity. To prevent the spread of powdery mildew infection, the leaves were treated after 6 weeks with Netz-Schwefelit WG (Neudorff), with carefully covered pots to prevent contact of the fungicide with the soil.

During the experiment, the chlorophyll, flavonol and anthocyanins concentration, as well as NBI (nitrogen balance index) in the leaves was measured with fluorescence using a leave-clip optical sensor (Dualex, Force-A, Orsay, France). After 8 weeks, plant growth parameters, including shoot length and shoot dry mass were measured in all 10 replicates. Fresh roots from 5 replicates were sampled, weighted, homogenized, and immediately frozen in liquid nitrogen for total phytoalexin content measurements. Remaining roots were weighted fresh and after drying, to extrapolate the total root dry mass. The soil adhering to the roots (rhizosphere) was brushed off with a sterile toothbrush and stored at -80 °C until DNA extraction.

### Phytoalexin quantification in roots by GC– MS

The total phytoalexin concentration in the roots was measured using gas chromatography-mass spectrometry (GC– MS) analysis. The frozen root samples were lyophilized and homogenized to a fine powder (29 Hz, 1 min; Mixer Mill MM400, Retsch, Haan, Germany). Phytoalexin extraction and quantification by GC– MS were performed according to the literature [13, 25, 26].

### DNA and library preparation for amplicon sequencing

DNA was extracted from 0.5 g of rhizosphere soil using phenol-chloroform extraction [45], and quantified using the Quant-iT PicoGreen dsDNA Assay Kit (Thermo Fisher Scientific, Darmstadt, Germany). To assess the bacterial community of the samples, the V4 region of the 16 S rRNA gene was amplified using the primer pair 515 F [46] and 806R [47]. All primers used in this study, plus respective sequences, are listed in Supplementary Table S1. The PCR reaction mix consisted of 12.5 µL NEBNext High-Fidelity 2x PCR Master Mix (New England Biolabs, Frankfurt am Main, Germany), 2.5 µL of 3% BSA, 0.3 µL of each 10 pmol primer, 17.5 ng of DNA template and DEPC-treated water up to 25 µL. The program for amplification was as follows: Initial denaturation for 1 min at 98 °C, followed by 30 cycles of 98 °C for 10 s denaturation, 55 °C for 30 s annealing, and 72 °C for 30 s elongation, with a final step for 5 min at 72 °C. The amplified product was purified using MagSi-NGSprep Plus beads (Steinbrenner, Wiesenbach, Germany). For Indexing PCR, a reaction mix consisting of 12.5 µL NEBNext HighFidelity 2 × PCR Master Mix, 1.5 µL of each indexing primer (Nextera^®^ XT Index Kit v2 Set B and D; Illumina, San Diego, CA, United States), 10 ng of the purified PCR product and DEPC-treated water up to 25 µL was used, with 98 °C for 30 s, 8 cycles of 98 °C for 10 s, 55 °C for 30s and 72 °C for 30 s, terminated by 72 °C for 5 min. The resulting amplicons were again purified using MagSi-NGSprep Plus beads, quantified, and quality checked using the Fragment Analyzer (Agilent Technologies, Santa Clara, CA, United States), and equimolarly pooled at 4 nM. For sequencing, the MiSeq^®^Reagent kit v3 (600 cycles) (Illumina, San Diego, CA, United States) was used for paired-end sequencing on an Illumina MiSeq^®^ instrument (Illumina, San Diego, CA, United States). The extraction of the negative controls (only sterile water) was run alongside the soil samples for all steps.

### Absolute quantification using qPCR

We quantified the bacterial 16 S rRNA gene and biphenyl dioxygenase genes (*bphd*) using quantitative real-time PCR (qPCR) of the extracted DNA. The reaction mix contained 12.5 µL *Power* SYBR™ Green PCR Master Mix (Applied Biosystems by Thermo Fisher Scientific, Darmstadt, Germany), 0.5 µL 3% BSA, 0.5 µL of each respective forward and reverse primer, 2 µL of DNA template and DEPC-treated water up to 25 µL. The primer pair used for 16 S rRNA gene qPCR was 16 S-f and 16 S-r [48]; for the *bphd* genes the primers BPHD-f3 and BPHD-r1 [49] (Supplementary Table S1) were implemented. The qPCR was run on a 7300 Real-Time PCR System (Applied Biosystems by Thermo Fisher Scientific, Darmstadt, Germany) with the following programs: For 16 S rRNA gene qPCR initial denaturation was done for 10 min at 95 °C, followed by 40 cycles of 95 °C for 45 s, 58 °C for 45 s and 72 °C for 45 s. It was terminated by a dissociation step at 95 °C for 15 s, 60 °C for 30 s, and 95 °C for 15 s. The program for *bphd* was the same, except for an annealing temperature of 57 °C. Amplification curves, melting temperature, and efficiency were checked with the 7300 System SDS Software v 1.3.1.21 (Applied Biosystems). Negative controls from the DNA and qPCR negative controls were included on the 96-well plates. The qPCR efficiency was calculated as E = − 1 + 10(− 1/slope) [50] and was 94.64% for 16 S rRNA and 88.89% for *bphd*. The coefficient of determination (R^2^) of the standard curves was determined to be above 0.99 for each qPCR. The specificity of the amplified products was checked with melting curves of the amplicons and on 1.5% agarose gels of randomly selected samples.

### Data processing and statistical analysis

The raw reads from Illumina sequences were processed on the Galaxy web server (www.usegalaxy.org) [51]. The adapter was trimmed from the FASTQ files, with a minimum read length of 50 using Cutadapt [52]. Read quality was controlled via FastQC [53]. For subsequent steps, the DADA2 pipeline (Galaxy Version 1.20) [54] was utilized with the following trimming and filtering parameters: 20 bp were cut terminally to remove the primer sequence and reads were truncated at position 240 (forward) and 190 (reverse), respectively, with an expected error of 3 (forward) and 4 (reverse). Taxonomy was assigned to the resulting unique amplicon sequencing variants (ASVs) using the SILVA database v138.1 release 99% [55]. ASVs assigned to mitochondrial and chloroplast reads, as well as ASVs assigned to reads from the negative controls and singletons, were removed from the dataset, to exclude potential contamination.

All following analyses were done in R version 4.3.1 [56]. The normality of the data and homogeneity of variances was checked using R-package tidyverse v 2.0.0 [57]. The 16 S rRNA gene dataset was analysed as a phyloseq object (R-package phyloseq v 1.46.0 [58]) and was normalized by scaling with ranked subsampling (SRS) [59]. If not noted otherwise, graphs were done using R-package ggplot2 v 3.5.0 [60]. Alpha diversity indices (Shannon diversity index, Pielou evenness) were calculated using the R-package microbiome v 1.24.0. Significant differences of alpha diversity indices and plant parameters were tested using the “compare_means” function of R-package ggpubr v 0.6.0 with method = wilcox.test and Bonferroni correction for multiple testing (*p* < 0.05). Beta diversity was assessed using Bray-Curtis dissimilarities and ordinated by non-metric multidimensional scaling (NMDS), as well as with principle coordinate analysis (PCoA), using R-package microViz v 0.12.1 [61]. Significant differences in community composition were tested with PERMANOVA (*p* < 0.05), using adonis2 function as implemented in R-package vegan v 2.6-4 and pairwise PERMANOVA using pairwise.adonis2 v 0.4.1. Relative abundance of the 30 most abundant genera was analysed using R-package ampvis2 v 2.7.33 [62], then visualized using the R-package tidyHeatmap v 1.10.1 [63]. A pairwise Wilcoxon test was used to analyse significant differences of the 30 most abundant genera between treatments. Boxplots of the top 9 genera were done with R-package microbiomeutilities v 1.00.17. Additionally, Venn diagrams of shared ASVs per treatment were created using InteractiVenn (https://www.interactivenn.net/) [64]. ASVs had to be detected in 4 of 5 replicates to be considered. Differential abundance and enrichment (log2-fold change) of taxa in the treatments were calculated using R-package DESeq2 v 1.42.1 [65] with default parameter and DESeq2 as implemented in the R-package microbiomeMarker v 1.8.0 [66]. Bacterial networks were constructed based on Spearman’s rank correlation from the R-package NetCoMi v 1.1.0 [67]. We used a centered log-ratio (clr) transformation for data normalization, handled zeros by pseudo count, and set a 0.3 correlation coefficient threshold as the sparsification method. A taxonomic heatmap of the top 50 genera connected as nodes to *R. pseudokoreensis* R79^T^ in the microbial networks IC8 and IC9 was constructed using the R-package microeco v 1.5.0 [68]. Partial correlation analysis was performed using the R-package corpcor v 1.6.10. The normalized phyloseq was filtered for ASVs that are present in all treatments, before performing the partial correlation analysis of ASVs against the inoculation load, which was handled as a continuous variable.

## Results

### Plant response and bacterial abundance at the plant soil interface

After 8 weeks of growth in the greenhouse, the root and shoot dry mass and root-to-shoot ratios were not significantly different, neither between the control and treatments, nor between inoculation treatments. Relative shoot growth (shoot length at sampling/shoot length at planting), was similar for the control, IC7 and IC8, and significantly higher than for plants from IC6 and IC9 (Supplementary Fig. S1). However, differences were small in general. From visual examination, the roots of all treatments showed typical ARD browning. Fluorescence measurements of leave chlorophyll concentration and other parameter (Supplementary Fig. S2) were not significantly different between the treatments in most cases due to large variance within the replicates. The detailed p-values can be found in Supplementary Table S2. Of the 10 replicates measured for plant growth parameters, 5 were randomly selected for further analysis.

Phytoalexin concentration in the roots was measured as an indicator for biotic stress in the plant, especially induced by ARD, and ranged from min 8.7 µg / g DM (dry mass) in the control to max 42.4 µg / g DM in IC8. There were no significant differences between inoculated treatments or the control, except for control vs. IC6 (Supplementary Fig. S3). Especially for IC8 and IC9, the variance among replicates appeared large, but lower/higher values did not correspond to better growth or higher/lower root or shoot biomass in the respective individual samples.

The overall bacterial abundance based on 16 S rRNA gene qPCR was lowest in control and IC6 samples and significantly higher in IC8 and IC9. The abundance in IC7 was on the same level as in IC8 and IC9, however, the variance was slightly too high for a significant difference to control / IC6 (Supplementary Fig. S4). The abundance of *bphd* genes increased with increasing inoculation concentration, which was significant only for IC9 compared to the control / IC6 / IC7 treatments.

### Changes in diversity of the bacterial rhizosphere community

**Fig. 1:**
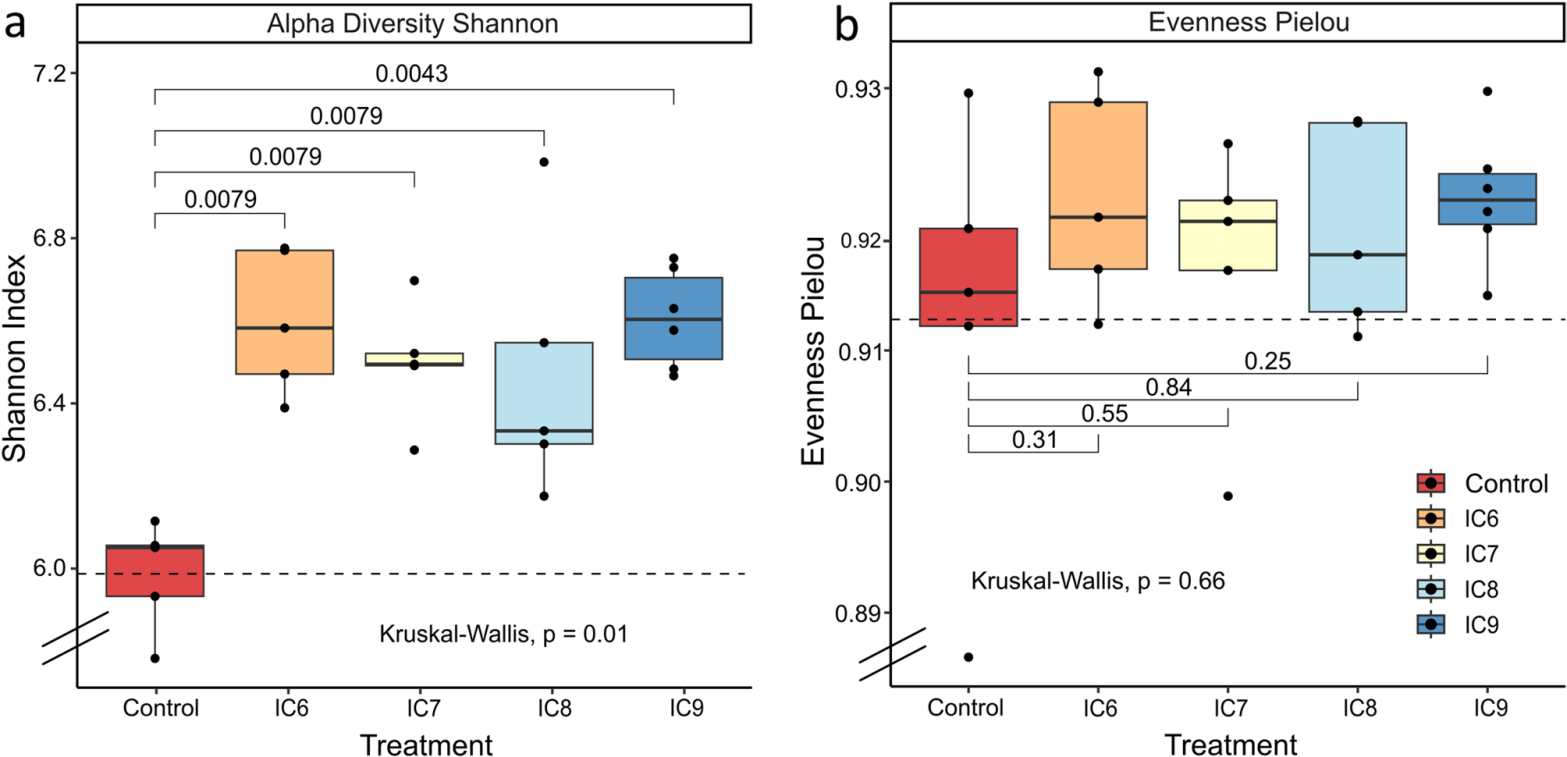
Comparison of alpha diversity indices of rhizosphere bacterial communities of apple plantlets inoculated with R. pseudokoreensis R79^T^ at different concentrations (**a**) Shannon Index and (**b**) Evenness Pielou. Significance was tested using Kruskal-Wallis and Wilcoxon Rank-Sum test with Bonferroni correction for multiple testing. Treatments are the uninoculated control and inoculated treatments from 2.5*10^6^ CFU/ml to 2.5*10^9^ CFU/ml (IC6 - IC9), *n* = 5

16S rRNA gene amplicon sequencing resulted in 2,090,470 high-quality reads. After filtering, denoising, merging, and bimera removal, 1,492,491 sequencing reads remained. The Shannon index of the rhizosphere samples was significantly enhanced by inoculation irrespective of the concentration compared to the control (*p* = 0.0079 for control vs. IC6– IC8, *p* = 0.0043 vs. IC9) (Fig. 1). The evenness was similar for control and inoculated samples, with again no visible influence of the different inoculation concentration.

**Fig. 2:**
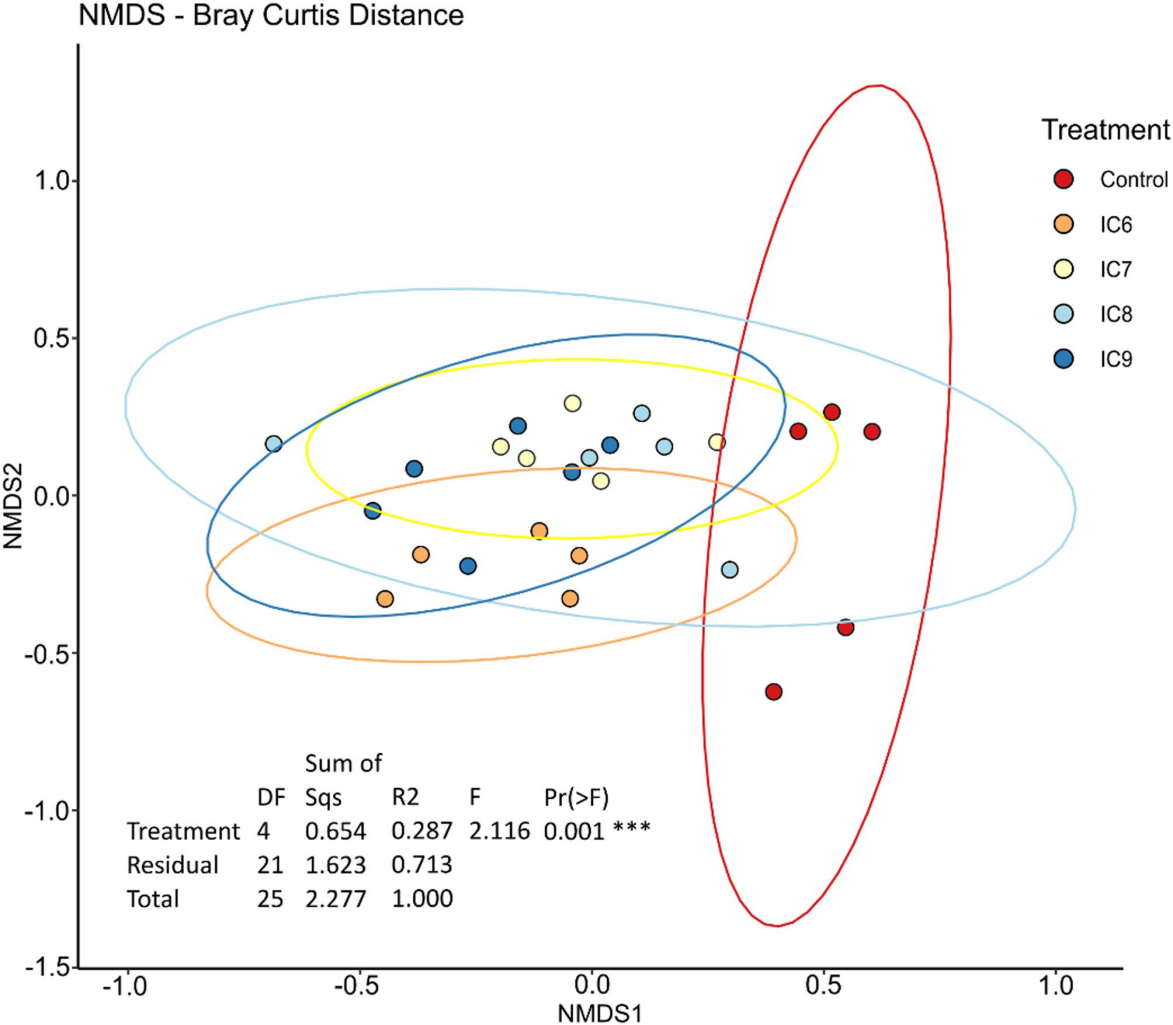
Bacterial community structure: NMDS plot based on Bray-Curtis distances showing the beta diversity of the samples. Treatments are the uninoculated control and inoculated treatments from 2.5*10^6^ CFU/ml to 2.5*10^9^ CFU/ml (IC6 - IC9). PERMANOVA showed the significant influence of the treatments on community composition

Inoculation treatments resulted in clusters clearly separated from the control cluster but not clearly separated from each other in an NMDS plot (Fig. 2). However, according to PERMANOVA, there were also significant differences between the different levels of inoculation (*p* < 0.001). This was the same when the control samples were excluded from the analysis. Here PERMANOVA showed a clear significant influence of the inoculation concentration on sample composition (*p* = 0.003), but still the separation of clusters based on NMDS was weak (Supplementary Fig. S5). Visually on NMDS axis 1 and 2, the inoculated samples did not follow a trendline corresponding to rising inoculation level, but the cluster had a more random distribution. The pairwise PERMANOVA (Supplementary Tables S3 and S4) showed significant differences for all combinations of treatments, except for IC7 vs. IC8 and IC8 vs. IC9, as well as IC8 vs. control.

### Community composition and differentially abundant taxa

**Fig. 3:**
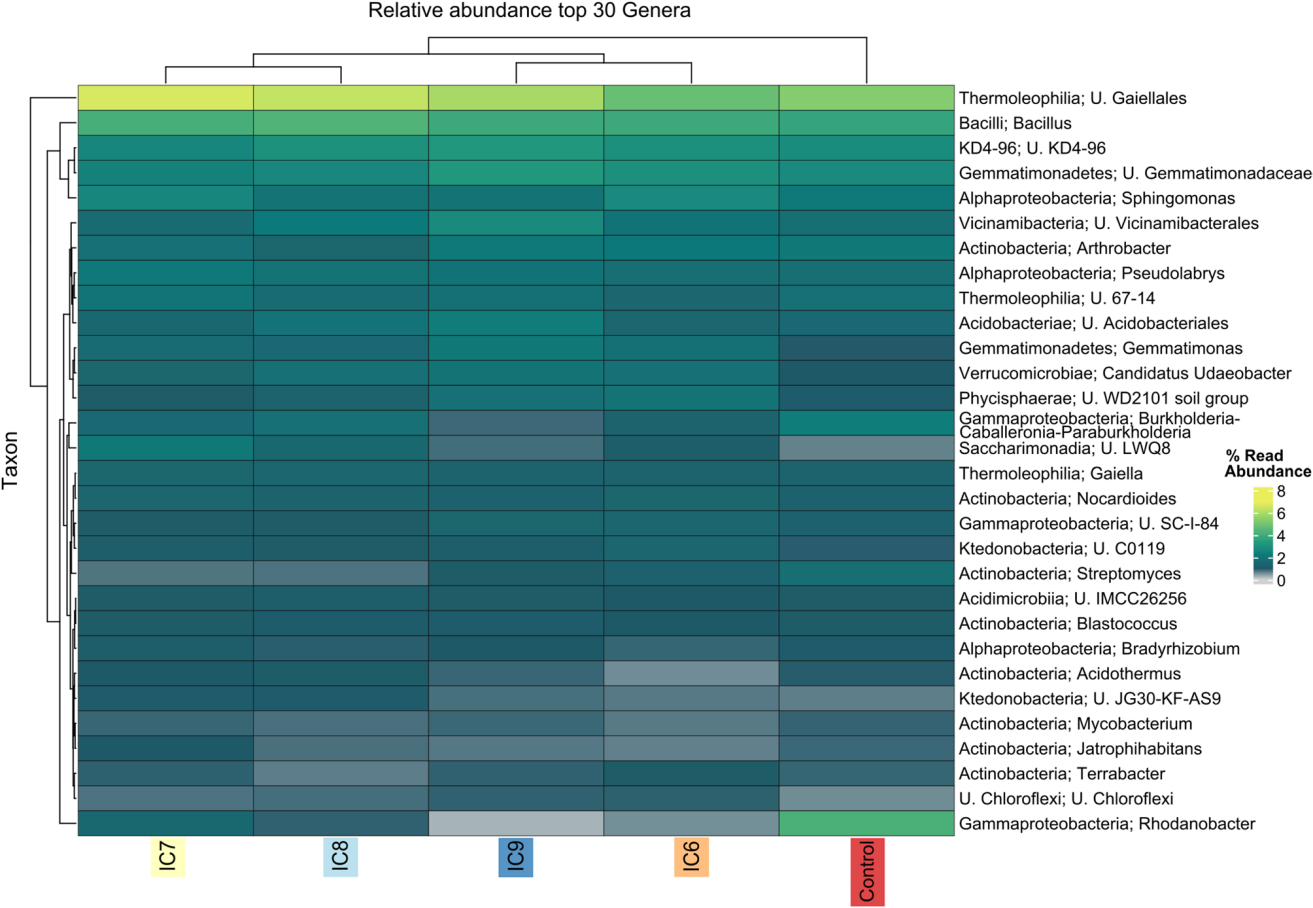
Taxonomic composition: Thirty most abundant genera with corresponding phylum names in percentage of relative abundance of the respective treatment. Treatments are the uninoculated control and inoculated treatments from 2.5*10^6^ CFU/ml to 2.5*10^9^ CFU/ml (IC6 - IC9). U. indicates an unknown member (genus) of this (higher) taxonomic group. Names consisting of numbers and letters are taxa belonging to groups without validly published scientific names, mostly coming from sequencing data. The heatmap was calculated and displayed using the R-package ampvis2 v.2.8.6 and tidyHeatmap v.1.10.1

After filtering and normalization, 4227 ASVs were recovered in the bacterial 16 S rRNA gene count table and 21.5% (904) of all ASVs were shared between all treatments. Following the trend of alpha diversity, the control had the least amount of unique ASVs (181 ASVs / 4.3%), followed by IC7, IC8, IC9 and IC6 with 277 ASVs / 6.6%, 346 ASVs / 8.2%, 359 ASVs / 8.6% and 444 ASVs / 10.6%, respectively (Supplementary Fig. S6 A/C). Considering only the inoculation treatments, 28.9% of ASVs were shared among them (Supplementary Fig. S6 B/D). The control treatment had the lowest number of ASVs shared with one of the other treatments; IC8 shared more ASVs with IC7 than with the others, and IC6 shared more ASVs with IC9. This similarity pattern of the communities was also mirrored by the hierarchical clustering of treatments in the dendrogram of Fig. 3, which further supported the observation of the beta diversity of the control being most distant to all inoculation treatments. Although clustering together in the dendrogram of Fig. 3, IC6 and IC9 differed significantly, as seen in the beta-diversity plot (Fig. 2). Among the top abundant genera were several Actinobacteria including *Arthrobacter*, *Nocardioides*, *Streptomyces*, *Blastococcus* and *Terrabacter* (Fig. 3), in addition to *Sphingomonas*, *Bacillus* and an unknown member of the Order Gaiellales. *Rhodanobacter* was noticeably higher in abundance in the control samples compared to the inoculated treatments. While rising inoculation concentration seemed to have triggered rising abundance of U. Acidobacteriales, U. KD4-96, *Pseudolabrys*, and U. Vicinamibacteriales, *Bacillus* abundance seemed to rise until inoculation treatment IC8, and then to decline again (Supplementary Fig. S7). According to the pairwise Wilcoxon test, only *Rhodanobacter* in IC9 was significantly different to the Control after Bonferroni correction for multiple testing (Supplementary Table S5).

**Fig. 4:**
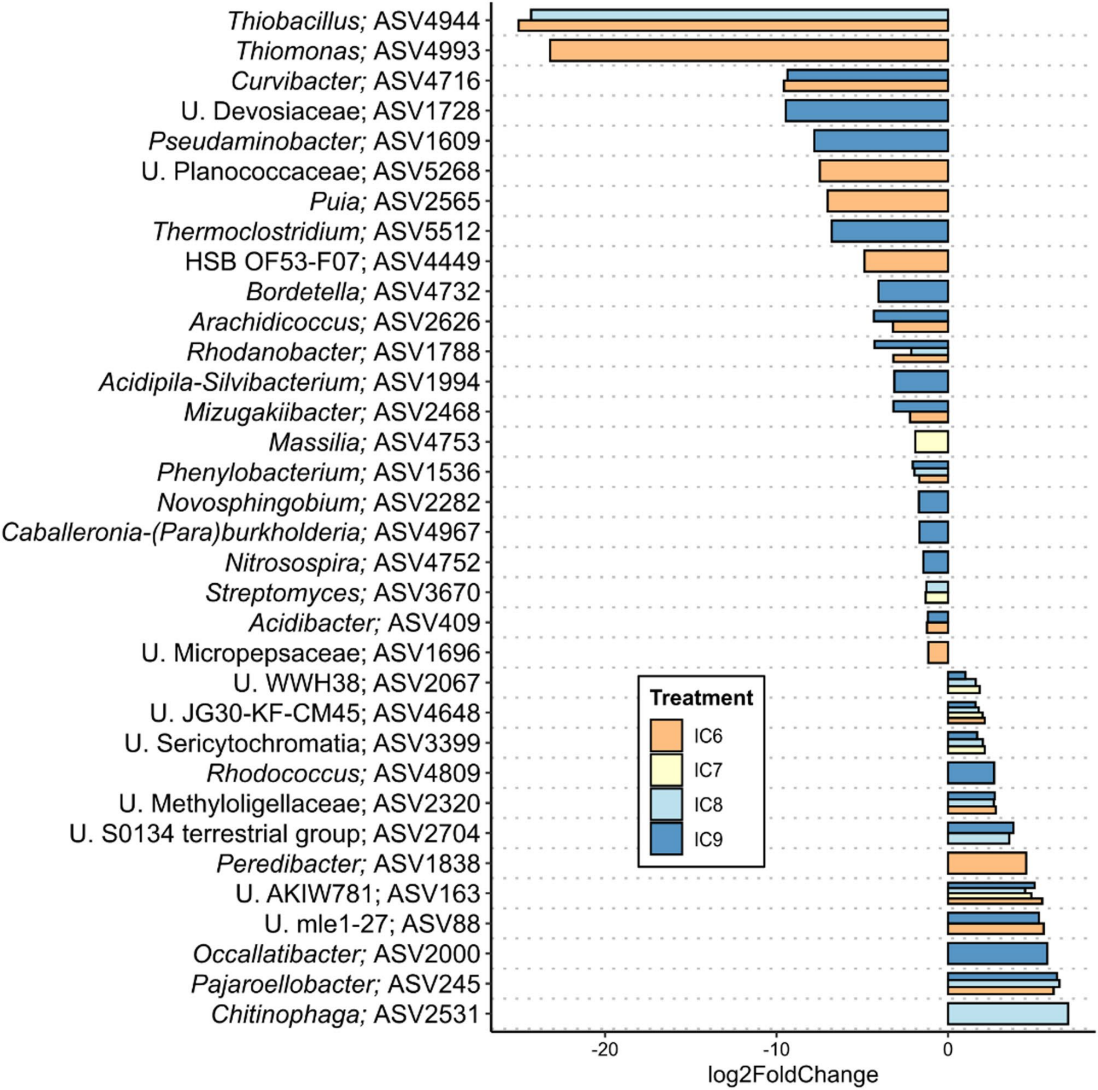
Significantly differentially abundant bacterial ASVs in the rhizosphere of apple plantlets after inoculation with different concentrations of R. pseudokoreensis R79^T^; inoculated samples (10^6^– 10^9^ CFU/ml = IC6– IC9) relative to the uninoculated control, log2-fold changes are shown on x-axis. Displayed are differentially abundant taxa with a p-value < 0.05

Taxa that were significantly differentially abundant between the uninoculated control and the respective different inoculation concentrations were identified using deseq2 (Fig. 4). The ASV corresponding to the inoculated *Rhodococcus* strain was found to be differentially higher abundant in IC9 compared to the control and additionally in IC8 using an additional tool, indicating a stable establishment of the strain during the experimental phase at least in treatments IC8 and IC9. As it was the only ASV identified as *Rhodococcus*, and aligned perfectly to the respective partial 16 S sequence, we treated it as *R. pseudokoreensis* R79^T^. Although the control samples were lower in alpha diversity and absolute abundance of bacteria, the number of significantly differentially higher abundant taxa was higher for the control compared to IC6 and IC9; however lower for IC7 and IC8 (Fig. 4, negative values on x-axis). The only taxa that were differentially abundant for all treatments vs. the control were U. AKIW781, belonging to phylum Chloroflexi, order Kallotenuales and U. JG30-KF-CM45, phylum Chloroflexi, order Thermomicrobiales. *Streptomyces* was significantly less abundant in IC7 and IC8 compared to the control. The highest log2-fold change was observed for *Thiomonas* and *Thiobacillus* for the control against IC6 and IC6/IC8, respectively, followed by *Curvibacter* for control against IC6/IC9 and an unknown Devosiaceae genus for control against IC9. Partial correlation analysis revealed taxa that responded to the increase/decrease in inoculation concentration (Supplementary Fig. S8). Among positively correlated ASVs were several members of the Order Gaiellales, a *Sphingomonas* ASV, and an unknown member of the Xanthobacteraceae, while members of the KD4-96 group and *Streptomyces* were among the negatively correlated taxa.

Co-occurrence networks using Spearman’s rank correlation on ASV level were used to compare the structure and organization of the microbial communities in the different treatments (Table 1). The differences between the respective treatments regarding the network parameter were low. However, the clustering coefficient (i.e., the degree to which the nodes tend to cluster together, which can imply the presence of sub-communities or functional groups) was the highest in the control treatment (0.77) and the lowest in IC9 (0.71). This was mirrored by the edge connectivity (i.e., indicates how interconnected the communities are). The modularity was > 0 for all treatments, implying a non-random distribution. Modularity and positive edge percentage (number of positive connections) peaked in IC7. The overall number of taxa was correlated to the alpha diversity of the treatments. The only network with hubs (i.e., nodes with high connectivity to others, possible keystone taxa) was the network of IC9 with a total of 37 detected hubs (Supplementary Table S6).

The inoculated *R. pseudokoreensis* R79^T^ was detected as member of the bacterial network in the treatments IC8 and IC9, with 518 and 628 edges (connected nodes), respectively. *R. pseudokoreensis* R79^T^ was not found in IC6 and IC7. The connected ASVs belonged mainly to taxa U. Gaiellales, *Sphingomonas*, *Pseudolabrys*, *Gemmatimonas*, U. KD4-96 and U. Vicinamibacteriales. *Arthrobacter*, the top abundant genus connected to *R. pseudokoreensis* R79^T^ in the IC9 network and *Bacillus*, the second most abundant genus connected to the inoculant in the IC8 network, were not present among the top 50 connected taxa of the respective other treatment (Supplementary Fig. S9).

**Table 1:** Parameters of the different bacterial networks constructed based on Spearman’s rank correlation. Networks are on ASV level. Treatments are the uninoculated control and inoculated treatments from 2.5*10^6^ CFU/ml to 2.5*10^9^ CFU/ml (IC6 - IC9)

Network	Clustering coefficient	Modularity	Positive edge percentage	Edge density	Natural connectivity	Vertex connectivity	Edge connectivity	Average dissimilarity	Average path length	Number of taxa
Control	0.77	0.09	54.09	0.62	0.24	137	137	0.80	1.15	511
IC6	0.72	0.14	54.04	0.55	0.19	381	397	0.83	1.17	1041
IC7	0.74	0.21	60.51	0.56	0.19	298	300	0.83	1.17	1006
IC8	0.76	0.16	56.14	0.59	0.24	249	252	0.81	1.16	900
IC9	0.71	0.09	58.41	0.61	0.15	493	493	0.87	1.13	1231

## Discussion

### Concentration depending inoculation success

The presence of DNA sequencing reads of *R. pseudokoreensis* R79^T^ in all treatments except the control (Supplementary Fig. S10), and their proportional increase to the respective inoculation concentration suggests the strain to be a successful colonizer in ARD soil. The absolute 16 S rRNA gene abundance indicated low levels for the control and IC6 and higher levels for IC7, IC8, and IC9, suggesting that survival of the inoculant at concentration 10^6^ CFU/ml was minimal, compared to the others. Our results are in line with a study about recurrent inoculation that describes a strong decline of a *Pseudomonas* inoculant with 10^6^ CFU/g soil until it was below the qPCR detection limit, while for inoculation with 10^8^ CFU/g soil, the abundance remained above 10^6^ CFU/g soil over the course of 14 weeks [40]. Additionally, the qPCR for *bphd* genes showed elevated abundance with rising inoculation concentration. As it is known that the biphenyl synthase expression in apple is correlated to ARD severity [69], it could be highly advantageous against ARD to have elevated levels of biphenyl degradation genes in the soil. Although it cannot be excluded that part of these data originate from resident DNA rather than from living cells [70], altogether our results suggest that at least for inoculation with IC8 and IC9 a significant number of cells were present in the rhizosphere after 8 weeks. Although we see improvement regarding the microbial diversity in soil for all inoculant concentrations, network parameters and the taxonomic composition might suggest a more stable state of the system at medium concentrations IC7 and IC8. As also the survival of inoculated cells has potentially been higher in IC8, the concentration of 10^8^ CFU/ml is suggested for further research in greenhouse experiments and field trials, to assess the effects of the inoculation at larger timescales and different soils.

### Inoculation with *R. pseudokoreensis* R79^T^ changes the co-occurrence pattern of microbial communities in the rhizosphere

Inoculation increased the diversity of rhizosphere communities and the structure of co-occurrence networks, with more positive interactions in inoculation treatments than in the controls and a higher modularity, particularly at IC7. Inoculation also resulted in stronger interconnected communities (higher edge connectivity). A non-zero modularity implies a non-random distribution, and higher modularity values might indicate more robust networks [71]. Hubs (nodes with a disproportionately high number of connections relative to their abundance) were only present in the co-occurrence network of IC9. Hubs can represent keystone species, which are usually defined as having a disproportionately large effect on the ecosystem relative to their abundance [72]. They impact network structure and stability through their interactions with others [73]. It implies that removing of those hubs might lead to drastic changes in the microbial networks / communities [74]. As most of the networks in our study did not display hubs, this might indicate communities that are evenly distributed and more resistant to change in case of node loss. However, interpretation is difficult, as the choice of tools alone will lead to immense differences in hub detection [75], and different to macro-ecological networks, co-occurrence in microbial networks does not necessarily imply a dependency on each other [74]. Our inoculant *R. pseudokoreensis* R79^T^ was part of the co-occurrence networks at concentrations of IC8 and IC9, even though it was not defined as one of the hubs. However, the number of interactions with other nodes was high, and the difference in the network structure of each treatment was evident. This indicated a lasting influence of inoculation on network structure, even at lower inoculation concentrations, where *R. pseudokoreensis* R79^T^ played no role in network structure. A similar effect was shown for the microbiome in root and adhering rhizosphere of banana inoculated with the biocontrol strain *Pseudomonas simiae* [7]. With inoculation, the topology of the network structure changed significantly compared to the control, even when the inoculant was not detected as a keystone taxon [7]. The integration of *R. pseudokoreensis* R79^T^ into the networks at higher concentrations suggested an active role of the inoculant in the microbial community. However, the network analyses should be interpreted cautiously and seen as a complement of the alpha and beta diversity measures. For more detailed network analysis, a high number of replicates might be necessary to gain enough robustness.

### Inoculation with *R. pseudokoreensis* R79^T^ enhances microbial diversity at the plant-soil interface

In our study, inoculation triggered a higher microbial diversity for all treatments, while evenness stayed the same, with no significant differences between the different inoculation concentrations. Several recent studies report positive effects of inoculation with different bacteria on diversity and the complexity of microbial networks in the rhizosphere, which were simultaneously associated with positive effects on plant performance [64, 65]. Also under abiotic stress, a *Rhodococcus* sp. strain inoculated into trace-metal contaminated soil stimulated a more biodiverse bacterial community and more complex bacterial networks [76]. Similarly for biotic stress, a high microbial diversity was shown to sustain soybean plant performance even at high levels of infection with root-lesion nematode *Pratylenchus* [77].

In our experiment, bacterial community structure was shifted for all concentrations in the same direction, with no visible succession following concentration load. Other recent works rarely studied the influence of different inoculant densities on the bacterial community composition. Overall, our data supported previous studies [40, 78] showing that inoculation density is an important factor in shaping community composition.

In our study, the most abundant bacterial genera mostly corresponded to taxa frequently described for apple rhizosphere, such as *Arthrobacter*, *Bacillus*, *Gemmatimonas*, *Sphingomonas*, and members of the Order *Gaiellales* [13, 42, 79]. The presence of potentially phenolic compound degrading bacteria (*Gemmatimonas*, *Mycobacterium* [80]; *Arthrobacter* [81]; *Sphingomonas* [82]) in the top abundant taxa was high and was not affected by inoculation. Inoculation with R79^T^ seemingly has not promoted a significantly higher abundance of prominent degraders of aromatic compounds. However, metagenome and transcriptome analysis are needed for further conclusions on abundance and expression of degradation abilities, which we cannot achieve with the resolution of a 16 S rRNA gene approach. In our study, a *Massilia* ASV was differentially higher abundant in the control compared to inoculation (IC7). This is in accordance with results from a reanalysis of several ARD and non-ARD datasets, where *Massilia* was enriched in ARD rhizosphere soil [83]. However, *Massilia* was also found at higher abundances in the rhizosphere of apple roots grown in the non-replant soil compartment in a split root experiment [19]. Given that members of *Massilia* were proposed as potential keystone taxa behind pathogen suppression in bacterial wilt disease in tomato [84], and if that is similar in apple, a reduction of *Massilia* after inoculation could interfere with disease suppression. However, we did not observe such negative effects in our plant growth data (see below). The genus *Streptomyces* was less abundant in the inoculated treatments and even significantly differentially less abundant in the treatments IC7 and IC8 compared to the control. Additionally, it was negatively correlated with inoculation load. We found this interesting, as *Streptomyces* ASVs were often associated with replant disease-affected soil in German orchards [13, 19, 30, 79] and were even negatively correlated to apple plant performance [85]. Mahnkopp-Dirks et al. (2022) showed endophytic *Streptomyces* to accumulate in roots grown in ARD soil, but over time also in roots grown in grass-control soil after planting of apple [86]. Thus, inoculation with R79^T^ could have a positive effect against a bacterium suggested to be associated with ARD.

### Effects on plant performance

Our analysis revealed no significant effects of inoculation on the overall plant growth after 8 weeks of growth in ARD soil in the greenhouse. This corresponds to other studies with small plants in relatively short-term experiments in the context of ARD, where inoculation with various bacteria resulted only in differences in root morphology [13], or was associated with inconsistent results in pot and field experiments [12]. Several studies with PGP inoculants, mainly *Bacillus* and arbuscular mycorrhizal fungal strains, reported enhanced growth of apple plantlets in greenhouse experiments [87–89], and fewer also found effects in field trials [90, 91]. However, *R. pseudokoreensis* R79^T^ was not expected to be an active PGP strain but to have a beneficial effect on the resident microbiome through its potential biphenyl degradation. The missing significant differences in plant growth might be due to several factors. While differences in apple plantlet growth were very well visible in greenhouse biotests with ARD and sterilized control soil after 8 weeks [92], possible growth differences between the different inoculation levels in the same ARD affected soil might be too subtle, especially compared to the in general high variability among the replicates. The possible effect of the changed microbiome on the plant performance might also need longer to show, and does not necessarily manifest in plant growth, but possibly also in better rejection of pathogens or higher resistance to biotic and abiotic stress. We saw similar plant performance under control and inoculated conditions and similar levels of phytoalexins in roots, not following the concentration of inoculation, although it must be noted that a study on apple rootstocks found phytoalexin profiles of rootstocks could be distinguished well 4 weeks after planting, but not after 8 weeks [93]. The low values differed from studies where inoculation induced massive increases in root phytoalexin content [13] and suggests R79^T^ as a possible candidate with low biotic stress potential on apple roots. Observed variance in the root phytoalexin content was not correlated to growth parameter of the individual replicates and can be attributed to the high natural variability of the plants. While the variance in root phytoalexin concentrations appeared relatively large, the absolute values were low and differences not significant, especially compared to measurements reported in studies such as Hauschild et al. [13]. However, in addition to plant growth parameters, transcriptional and metabolic analysis are needed and would allow to identify plant responses and evaluate inoculation effects on the plant side in more detail.

*R. pseudokoreensis* R79^T^ grew in vitro in the presence of several biphenyl phytoalexins extracted from apple roots at higher levels than in the environment before getting inhibited [27]. This aligns with its genetic potential to degrade biphenyls [32]. Although the growth of R79^T^ was severely affected by dibenzofurans, another class of apple phytoalexins, in an in vitro assay [27], our results based on ASV counts and abundance of biphenyl degradation genes suggested that *R. pseudokoreensis* R79^T^ can tolerate phytoalexin concentrations in ARD-affected apple rhizosphere. As hypothesized, inoculation with R79^T^ enhanced microbial diversity and strengthened network structures in the rhizosphere in ARD soil. It also did not pose any additional stress on the plantlets. However, our study design could not resolve if the enhanced diversity is a direct beneficial effect based on modulating the chemical composition of the rhizosphere due to biphenyl degradation or if this effect might extend beyond direct phytoalexin degradation. Duan et al. (2022) showed that a plant growth-promoting and biocontrol active strain of *Bacillus licheniformis* was able to promote apple plant growth and reduce the number of pathogens in replant soil, while also reducing the amount of general phenolic compounds, especially phlorizin in soil [90]. While opposite to our study, the focus of their study lay on PGP and biocontrol and not on microbiome changes, the approach to alleviate ARD through changing the soil microbiome using degradation of aromatic compounds / phytoalexins in soil seems promising. However, transcriptomics and metabolomics data are needed to provide a clearer understanding of the molecular processes in the rhizosphere, and if the degradation of aromatic compounds actually played a role in the bacterial community changes observed in this study. Field experiments are needed to assess the inoculant’s performance in the long term, as well as in experiments with inoculation of the soil prior to planting, to potentially prepare ARD affected soil for of young apple plantlets.

## Conclusion

The present study demonstrated the successful inoculation of *R. pseudokoreensis* R79^T^ on young apple plantlets grown in ARD soil. It showed a positive influence of the inoculant on microbiome diversity and no negative influence of inoculation on plant performance after 8 weeks. This work provides critical insights into the strain’s measurable effects under controlled conditions and provides a base for subsequent field trials and the use in microbial consortia, where the observed effects can be further validated under more complex and variable environmental conditions. It is important to note that this study was not designed to be able to separate the direct effects of the inoculant from indirect effects mediated through plant responses or subsequent microbiome changes. The next steps need to involve transcriptomic and metabolomic analysis to provide mechanistic understanding of the increase in bacterial diversity, the microbial functions, and if the increase is connected to aromatic compound degradation in ARD soil. Highly controlled experiments with knock-out mutants of R79^T^ are necessary to prove the degradation of biphenyls as a mechanistic effect. Furthermore, it is crucial to test if the elevated diversity leads to improved plant performance in ARD soil in the long term. For inoculation with R79^T^ at field scale and on different soils, a concentration of 10^8^ CFU/ml appears most appropriate. For elevated effectiveness against ARD symptoms *R. pseudokoreensis* R79^T^ should be applied as part of a consortium to combine its positive effects on biodiversity with other bacteria known to enhance growth, nutrient availability, and pathogen control. Large-scale field trials within the ORDIAmur project are currently ongoing, testing *R. pseudokoreensis* R79^T^ in a consortium with a *Bacillus* and a *Rhizophagus* strain. The idea of mitigating ARD by influencing the soil microbiome through degrading phytoalexins is a new bioinoculation approach, and it still needs to be proven if the degradation of phytoalexins can improve apple plant performance in the long term. Moreover, additional research is needed on the actual role of phytoalexins in the development of ARD. Introducing phytoalexin degraders also might need to be balanced against the risk to deprive plants of the antimicrobial effect of their phytoalexins, particularly when expanding such an approach to crops other than apple. Still, as replant disease is also known for other members of the Rosaceae, expansion of the concept to other plants might be possible.

## Electronic supplementary material

Below is the link to the electronic supplementary material.

Supplementary Material 1

## Data Availability

The nucleotide sequence data reported here are available as raw reads in the Sequence Read Archive (SRA) of NCBI database under the BioProject PRJNA700828 (https://www.ncbi.nlm.nih.gov/sra/PRJNA700828), accession numbers SAMN43521822 to SAMN43521851. The scripts for the bioinformatic analysis can be retrieved from GitHub under https://github.com/sarahmabenning/Inoculation_R79T.
