# Supplementary material for: Inoculation of apple plantlets with Rhodococcus pseudokoreensis R79T enhances diversity and modulates the structure of bacterial rhizosphere communities in soil affected by apple replant disease
Source: BMC Plant Biol. 2025 May 28;25:715. doi: 10.1186/s12870-025-06747-9 (PMC12117973; doi:10.1186/s12870-025-06747-9)
Supplement: Supplementary file 1 — Supplementary Material 1 [file 12870_2025_6747_MOESM1_ESM.pdf]

Supplementary file for Inoculation of apple plantlets with *Rhodococcus pseudokoreensis* R79^T enhances diversity and modulates the structure of bacterial rhizosphere communities in soil affected by apple replant disease

Sarah Benning[a]#, Fatma M. Mahmoud[ab], Pamela Espindola-Hernandez[a], Benye Liu[c], Karin Pritsch[d], Viviane Radl[a]*, Jana Barbro Winkler[d], Traud Winkelmann[e], Ludger Beerhues[b], Michael Schloter[f,a]

[a] Research Unit for Comparative Microbiome Analysis, Helmholtz Munich, German Research Center for Environmental Health, Neuherberg, Germany

[b] Botany and Microbiology Department, Faculty of Science, Suez Canal University, Ismailia, Egypt

[c] Institute of Pharmaceutical Biology, Technische Universität Braunschweig, Germany

[d] Research Unit for Environmental Simulations, Helmholtz Munich, German Research Center for Environmental Health, Neuherberg, Germany

[e] Institute of Horticultural Production Systems, Leibniz University Hannover, Germany

[f] Chair for Environmental Microbiology, TUM School of Life Sciences, Technical University Munich, Germany

Running Head: Inoculation study with R79^T

#Address correspondence to Sarah Benning, sarah.benning@helmholtz-munich.de

*Present address: Viviane Radl, Department of Safety and Area Management, Helmholtz Munich, German Research Center for Environmental Health, Neuherberg, Germany

Supplementary Tables

Supplementary Table S1. Sequencing and qPCR primers used in this study

Primer Name	Sequence 5’ – 3’	Gene	Amplification	Reference
515F	GTGYCAGCMGCCGCGGTAA	16S rRNA – V4	PCR	Parada et al. 2016 (1)
806R	GGACTACNVGGGTWTCTAAT	16S rRNA – V4	PCR	Apprill et al. 2015 (2)
16S-f	GGTAGTCYAYGCMSTAAACG	16S rRNA – V4	qPCR	Bach et al. 2002 (3)
16S-r	GACARCCATGCASCACCTG	16S rRNA – V4	qPCR	Bach et al. 2002 (3)
BPHD-f3	AACTGGAARTTYGCIGCVGA	bphd	qPCR	Iwai et al. 2010 (4)
BPHD-r1	ACCCAGTTYTCICCR TCGTC	bphd	qPCR	Iwai et al. 2010 (4)

Supplementary Table S2. Wilcoxon test results separated by timepoint (date) for Supplementary Figure S2, fluorescence measurements of chlorophyll (chl), flavonols (flav), anthocyanins (anth) and nitrogen balance index (nbi) in young apple plantlet leaves during 8 weeks growth in ARD soil in the greenhouse. Treatments are the uninoculated control and inoculated treatments from 2.5*10^6 CFU/mL to 2.5*10^9 CFU/mL (IC6 - IC9).

DATE	VARIABLE	GROUP1	GROUP2	P	P.ADJ	P.FORMAT	P.SIGNIF	METHOD
**04.05.2021**	chl	Control	IC6	0,73936435	1	0,73936	ns	Wilcoxon
**04.05.2021**	chl	Control	IC7	0,63052891	1	0,63053	ns	Wilcoxon
**04.05.2021**	chl	Control	IC8	0,06301284	1	0,06301	ns	Wilcoxon
**04.05.2021**	chl	Control	IC9	0,63052891	1	0,63053	ns	Wilcoxon
**04.05.2021**	chl	IC6	IC7	0,68421053	1	0,68421	ns	Wilcoxon
**04.05.2021**	chl	IC6	IC8	0,10512243	1	0,10512	ns	Wilcoxon
**04.05.2021**	chl	IC6	IC9	0,39304813	1	0,39305	ns	Wilcoxon
**04.05.2021**	chl	IC7	IC8	0,21756262	1	0,21756	ns	Wilcoxon
**04.05.2021**	chl	IC7	IC9	0,73936435	1	0,73936	ns	Wilcoxon
**04.05.2021**	chl	IC8	IC9	0,27986101	1	0,27986	ns	Wilcoxon
**11.05.2021**	chl	Control	IC6	0,35268137	1	0,35268	ns	Wilcoxon
**11.05.2021**	chl	Control	IC7	0,48125095	1	0,48125	ns	Wilcoxon
**11.05.2021**	chl	Control	IC8	0,63052891	1	0,63053	ns	Wilcoxon
**11.05.2021**	chl	Control	IC9	0,14314014	1	0,14314	ns	Wilcoxon
**11.05.2021**	chl	IC6	IC7	0,08920955	1	0,08921	ns	Wilcoxon
**11.05.2021**	chl	IC6	IC8	0,06301284	1	0,06301	ns	Wilcoxon
**11.05.2021**	chl	IC6	IC9	0,00287947	0,17	0,00288	**	Wilcoxon
**11.05.2021**	chl	IC7	IC8	0,73936435	1	0,73936	ns	Wilcoxon
**11.05.2021**	chl	IC7	IC9	0,52884886	1	0,52885	ns	Wilcoxon
**11.05.2021**	chl	IC8	IC9	0,27986101	1	0,27986	ns	Wilcoxon
**18.05.2021**	chl	Control	IC6	0,91179718	1	0,9118	ns	Wilcoxon
**18.05.2021**	chl	Control	IC7	0,43587218	1	0,43587	ns	Wilcoxon
**18.05.2021**	chl	Control	IC8	0,06301284	1	0,06301	ns	Wilcoxon
**18.05.2021**	chl	Control	IC9	0,10512243	1	0,10512	ns	Wilcoxon
**18.05.2021**	chl	IC6	IC7	0,68421053	1	0,68421	ns	Wilcoxon
**18.05.2021**	chl	IC6	IC8	0,10512243	1	0,10512	ns	Wilcoxon
**18.05.2021**	chl	IC6	IC9	0,08920955	1	0,08921	ns	Wilcoxon
**18.05.2021**	chl	IC7	IC8	0,12300548	1	0,12301	ns	Wilcoxon
**18.05.2021**	chl	IC7	IC9	0,21756262	1	0,21756	ns	Wilcoxon
**18.05.2021**	chl	IC8	IC9	0,97051246	1	0,97051	ns	Wilcoxon
**25.05.2021**	chl	Control	IC6	0,12300548	1	0,12301	ns	Wilcoxon
**25.05.2021**	chl	Control	IC7	0,16549395	1	0,16549	ns	Wilcoxon
**25.05.2021**	chl	Control	IC8	0,68421053	1	0,68421	ns	Wilcoxon
**25.05.2021**	chl	Control	IC9	0,31499924	1	0,315	ns	Wilcoxon
**25.05.2021**	chl	IC6	IC7	0,73936435	1	0,73936	ns	Wilcoxon
**25.05.2021**	chl	IC6	IC8	0,43587218	1	0,43587	ns	Wilcoxon
**25.05.2021**	chl	IC6	IC9	0,19031588	1	0,19032	ns	Wilcoxon
**25.05.2021**	chl	IC7	IC8	0,39304813	1	0,39305	ns	Wilcoxon
**25.05.2021**	chl	IC7	IC9	0,24745069	1	0,24745	ns	Wilcoxon
**25.05.2021**	chl	IC8	IC9	0,97051246	1	0,97051	ns	Wilcoxon
**08.06.2021**	chl	Control	IC6	0,00072528	0,04	0,00073	***	Wilcoxon
**08.06.2021**	chl	Control	IC7	0,12300548	1	0,12301	ns	Wilcoxon
**08.06.2021**	chl	Control	IC8	0,27986101	1	0,27986	ns	Wilcoxon

**08.06.2021**	chl	Control	IC9	0,03546299	1	0,03546	*	Wilcoxon
**08.06.2021**	chl	IC6	IC7	0,01468964	0,88	0,01469	*	Wilcoxon
**08.06.2021**	chl	IC6	IC8	0,0089307	0,54	0,00893	**	Wilcoxon
**08.06.2021**	chl	IC6	IC9	0,52884886	1	0,52885	ns	Wilcoxon
**08.06.2021**	chl	IC7	IC8	0,52884886	1	0,52885	ns	Wilcoxon
**08.06.2021**	chl	IC7	IC9	0,0524259	1	0,05243	ns	Wilcoxon
**08.06.2021**	chl	IC8	IC9	0,14314014	1	0,14314	ns	Wilcoxon
**22.06.2021**	chl	Control	IC6	0,22953517	1	0,22954	ns	Wilcoxon
**22.06.2021**	chl	Control	IC7	0,57874169	1	0,57874	ns	Wilcoxon
**22.06.2021**	chl	Control	IC8	0,57874169	1	0,57874	ns	Wilcoxon
**22.06.2021**	chl	Control	IC9	0,73961333	1	0,73961	ns	Wilcoxon
**22.06.2021**	chl	IC6	IC7	0,22953517	1	0,22954	ns	Wilcoxon
**22.06.2021**	chl	IC6	IC8	0,31478815	1	0,31479	ns	Wilcoxon
**22.06.2021**	chl	IC6	IC9	0,12820513	1	0,12821	ns	Wilcoxon
**22.06.2021**	chl	IC7	IC8	0,85342831	1	0,85343	ns	Wilcoxon
**22.06.2021**	chl	IC7	IC9	0,31478815	1	0,31479	ns	Wilcoxon
**22.06.2021**	chl	IC8	IC9	0,60088441	1	0,60088	ns	Wilcoxon
**04.05.2021**	flav	Control	IC6	0,27986101	1	0,27986	ns	Wilcoxon
**04.05.2021**	flav	Control	IC7	0,04325705	1	0,04326	*	Wilcoxon
**04.05.2021**	flav	Control	IC8	0,06301284	1	0,06301	ns	Wilcoxon
**04.05.2021**	flav	Control	IC9	0,02830604	1	0,02831	*	Wilcoxon
**04.05.2021**	flav	IC6	IC7	0,57874169	1	0,57874	ns	Wilcoxon
**04.05.2021**	flav	IC6	IC8	0,49612971	1	0,49613	ns	Wilcoxon
**04.05.2021**	flav	IC6	IC9	0,27986101	1	0,27986	ns	Wilcoxon
**04.05.2021**	flav	IC7	IC8	0,97051246	1	0,97051	ns	Wilcoxon
**04.05.2021**	flav	IC7	IC9	0,63052891	1	0,63053	ns	Wilcoxon
**04.05.2021**	flav	IC8	IC9	0,73936435	1	0,73936	ns	Wilcoxon
**11.05.2021**	flav	Control	IC6	0,14314014	1	0,14314	ns	Wilcoxon
**11.05.2021**	flav	Control	IC7	0,00219376	0,13	0,00219	**	Wilcoxon
**11.05.2021**	flav	Control	IC8	0,01468964	0,88	0,01469	*	Wilcoxon
**11.05.2021**	flav	Control	IC9	7,58E-05	0	7,60E-05	****	Wilcoxon
**11.05.2021**	flav	IC6	IC7	0,0072626	0,44	0,00726	**	Wilcoxon
**11.05.2021**	flav	IC6	IC8	0,06301284	1	0,06301	ns	Wilcoxon
**11.05.2021**	flav	IC6	IC9	0,0001299	0,01	0,00013	***	Wilcoxon
**11.05.2021**	flav	IC7	IC8	0,07555159	1	0,07555	ns	Wilcoxon
**11.05.2021**	flav	IC7	IC9	0,14031583	1	0,14032	ns	Wilcoxon
**11.05.2021**	flav	IC8	IC9	0,00150469	0,09	0,0015	**	Wilcoxon
**18.05.2021**	flav	Control	IC6	0,52884886	1	0,52885	ns	Wilcoxon
**18.05.2021**	flav	Control	IC7	7,58E-05	0	7,60E-05	****	Wilcoxon
**18.05.2021**	flav	Control	IC8	0,01468964	0,88	0,01469	*	Wilcoxon
**18.05.2021**	flav	Control	IC9	4,33E-05	0	4,30E-05	****	Wilcoxon
**18.05.2021**	flav	IC6	IC7	0,00020568	0,01	0,00021	***	Wilcoxon
**18.05.2021**	flav	IC6	IC8	0,02323064	1	0,02323	*	Wilcoxon
**18.05.2021**	flav	IC6	IC9	0,00020568	0,01	0,00021	***	Wilcoxon
**18.05.2021**	flav	IC7	IC8	0,11227551	1	0,11228	ns	Wilcoxon
**18.05.2021**	flav	IC7	IC9	0,07525601	1	0,07526	ns	Wilcoxon
**18.05.2021**	flav	IC8	IC9	0,02323064	1	0,02323	*	Wilcoxon
**25.05.2021**	flav	Control	IC6	0,52884886	1	0,52885	ns	Wilcoxon

**25.05.2021**	flav	Control	IC7	0,00058006	0,04	0,00058	***	Wilcoxon
**25.05.2021**	flav	Control	IC8	0,35268137	1	0,35268	ns	Wilcoxon
**25.05.2021**	flav	Control	IC9	0,00072528	0,04	0,00073	***	Wilcoxon
**25.05.2021**	flav	IC6	IC7	0,00058006	0,04	0,00058	***	Wilcoxon
**25.05.2021**	flav	IC6	IC8	0,04325705	1	0,04326	*	Wilcoxon
**25.05.2021**	flav	IC6	IC9	0,00020568	0,01	0,00021	***	Wilcoxon
**25.05.2021**	flav	IC7	IC8	0,01129895	0,68	0,0113	*	Wilcoxon
**25.05.2021**	flav	IC7	IC9	0,27285597	1	0,27286	ns	Wilcoxon
**25.05.2021**	flav	IC8	IC9	0,01468964	0,88	0,01469	*	Wilcoxon
**08.06.2021**	flav	Control	IC6	1,08E-05	0	1,10E-05	****	Wilcoxon
**08.06.2021**	flav	Control	IC7	0,0001299	0,01	0,00013	***	Wilcoxon
**08.06.2021**	flav	Control	IC8	0,00018165	0,01	0,00018	***	Wilcoxon
**08.06.2021**	flav	Control	IC9	1,08E-05	0	1,10E-05	****	Wilcoxon
**08.06.2021**	flav	IC6	IC7	0,57874169	1	0,57874	ns	Wilcoxon
**08.06.2021**	flav	IC6	IC8	0,52036602	1	0,52037	ns	Wilcoxon
**08.06.2021**	flav	IC6	IC9	0,07525601	1	0,07526	ns	Wilcoxon
**08.06.2021**	flav	IC7	IC8	0,73363364	1	0,73363	ns	Wilcoxon
**08.06.2021**	flav	IC7	IC9	0,35268137	1	0,35268	ns	Wilcoxon
**08.06.2021**	flav	IC8	IC9	0,47250907	1	0,47251	ns	Wilcoxon
**22.06.2021**	flav	Control	IC6	0,0185109	1	0,01851	*	Wilcoxon
**22.06.2021**	flav	Control	IC7	0,00519604	0,31	0,0052	**	Wilcoxon
**22.06.2021**	flav	Control	IC8	0,24745069	1	0,24745	ns	Wilcoxon
**22.06.2021**	flav	Control	IC9	0,05532703	1	0,05533	ns	Wilcoxon
**22.06.2021**	flav	IC6	IC7	0,96225833	1	0,96226	ns	Wilcoxon
**22.06.2021**	flav	IC6	IC8	0,88677499	1	0,88677	ns	Wilcoxon
**22.06.2021**	flav	IC6	IC9	0,02622378	1	0,02622	*	Wilcoxon
**22.06.2021**	flav	IC7	IC8	0,97051246	1	0,97051	ns	Wilcoxon
**22.06.2021**	flav	IC7	IC9	0,01357466	0,81	0,01357	*	Wilcoxon
**22.06.2021**	flav	IC8	IC9	0,07023858	1	0,07024	ns	Wilcoxon
**04.05.2021**	anth	Control	IC6	0,39304813	1	0,39305	ns	Wilcoxon
**04.05.2021**	anth	Control	IC7	0,43587218	1	0,43587	ns	Wilcoxon
**04.05.2021**	anth	Control	IC8	0,49596757	1	0,49597	ns	Wilcoxon
**04.05.2021**	anth	Control	IC9	0,13013598	1	0,13014	ns	Wilcoxon
**04.05.2021**	anth	IC6	IC7	0,04499303	1	0,04499	*	Wilcoxon
**04.05.2021**	anth	IC6	IC8	0,05859166	1	0,05859	ns	Wilcoxon
**04.05.2021**	anth	IC6	IC9	0,00812702	0,49	0,00813	**	Wilcoxon
**04.05.2021**	anth	IC7	IC8	0,79110651	1	0,79111	ns	Wilcoxon
**04.05.2021**	anth	IC7	IC9	0,19859576	1	0,1986	ns	Wilcoxon
**04.05.2021**	anth	IC8	IC9	0,32538732	1	0,32539	ns	Wilcoxon
**11.05.2021**	anth	Control	IC6	0,14314014	1	0,14314	ns	Wilcoxon
**11.05.2021**	anth	Control	IC7	0,06392213	1	0,06392	ns	Wilcoxon
**11.05.2021**	anth	Control	IC8	0,90962034	1	0,90962	ns	Wilcoxon
**11.05.2021**	anth	Control	IC9	0,00314009	0,19	0,00314	**	Wilcoxon
**11.05.2021**	anth	IC6	IC7	0,00512544	0,31	0,00513	**	Wilcoxon
**11.05.2021**	anth	IC6	IC8	0,0374199	1	0,03742	*	Wilcoxon
**11.05.2021**	anth	IC6	IC9	0,00020986	0,01	0,00021	***	Wilcoxon
**11.05.2021**	anth	IC7	IC8	0,25540492	1	0,2554	ns	Wilcoxon
**11.05.2021**	anth	IC7	IC9	0,54489574	1	0,5449	ns	Wilcoxon

**11.05.2021**	anth	IC8	IC9	0,01888481	1	0,01888	*	Wilcoxon
**18.05.2021**	anth	Control	IC6	0,70524564	1	0,70525	ns	Wilcoxon
**18.05.2021**	anth	Control	IC7	0,05849666	1	0,0585	ns	Wilcoxon
**18.05.2021**	anth	Control	IC8	0,34397759	1	0,34398	ns	Wilcoxon
**18.05.2021**	anth	Control	IC9	0,04102137	1	0,04102	*	Wilcoxon
**18.05.2021**	anth	IC6	IC7	0,13956907	1	0,13957	ns	Wilcoxon
**18.05.2021**	anth	IC6	IC8	0,73315031	1	0,73315	ns	Wilcoxon
**18.05.2021**	anth	IC6	IC9	0,10332876	1	0,10333	ns	Wilcoxon
**18.05.2021**	anth	IC7	IC8	0,095177	1	0,09518	ns	Wilcoxon
**18.05.2021**	anth	IC7	IC9	1	1	100.000	ns	Wilcoxon
**18.05.2021**	anth	IC8	IC9	0,06362233	1	0,06362	ns	Wilcoxon
**25.05.2021**	anth	Control	IC6	0,34415946	1	0,34416	ns	Wilcoxon
**25.05.2021**	anth	Control	IC7	0,90965423	1	0,90965	ns	Wilcoxon
**25.05.2021**	anth	Control	IC8	0,85342831	1	0,85343	ns	Wilcoxon
**25.05.2021**	anth	Control	IC9	0,84988351	1	0,84988	ns	Wilcoxon
**25.05.2021**	anth	IC6	IC7	0,28919212	1	0,28919	ns	Wilcoxon
**25.05.2021**	anth	IC6	IC8	0,34452278	1	0,34452	ns	Wilcoxon
**25.05.2021**	anth	IC6	IC9	0,34434119	1	0,34434	ns	Wilcoxon
**25.05.2021**	anth	IC7	IC8	1	1	100.000	ns	Wilcoxon
**25.05.2021**	anth	IC7	IC9	0,67712645	1	0,67713	ns	Wilcoxon
**25.05.2021**	anth	IC8	IC9	0,5703153	1	0,57032	ns	Wilcoxon
**08.06.2021**	anth	Control	IC6	0,03546299	1	0,03546	*	Wilcoxon
**08.06.2021**	anth	Control	IC7	0,91179718	1	0,9118	ns	Wilcoxon
**08.06.2021**	anth	Control	IC8	0,52884886	1	0,52885	ns	Wilcoxon
**08.06.2021**	anth	Control	IC9	0,16549395	1	0,16549	ns	Wilcoxon
**08.06.2021**	anth	IC6	IC7	0,01149624	0,69	0,0115	*	Wilcoxon
**08.06.2021**	anth	IC6	IC8	0,00519604	0,31	0,0052	**	Wilcoxon
**08.06.2021**	anth	IC6	IC9	0,48125095	1	0,48125	ns	Wilcoxon
**08.06.2021**	anth	IC7	IC8	0,59656316	1	0,59656	ns	Wilcoxon
**08.06.2021**	anth	IC7	IC9	0,0524259	1	0,05243	ns	Wilcoxon
**08.06.2021**	anth	IC8	IC9	0,0524259	1	0,05243	ns	Wilcoxon
**22.06.2021**	anth	Control	IC6	0,03169018	1	0,03169	*	Wilcoxon
**22.06.2021**	anth	Control	IC7	0,12300548	1	0,12301	ns	Wilcoxon
**22.06.2021**	anth	Control	IC8	0,10512243	1	0,10512	ns	Wilcoxon
**22.06.2021**	anth	Control	IC9	0,96225833	1	0,96226	ns	Wilcoxon
**22.06.2021**	anth	IC6	IC7	0,55794328	1	0,55794	ns	Wilcoxon
**22.06.2021**	anth	IC6	IC8	0,88677499	1	0,88677	ns	Wilcoxon
**22.06.2021**	anth	IC6	IC9	0,07284382	1	0,07284	ns	Wilcoxon
**22.06.2021**	anth	IC7	IC8	1	1	100.000	ns	Wilcoxon
**22.06.2021**	anth	IC7	IC9	0,10880296	1	0,1088	ns	Wilcoxon
**22.06.2021**	anth	IC8	IC9	0,13307281	1	0,13307	ns	Wilcoxon
**04.05.2021**	nbi	Control	IC6	0,12300548	1	0,12301	ns	Wilcoxon
**04.05.2021**	nbi	Control	IC7	0,16549395	1	0,16549	ns	Wilcoxon
**04.05.2021**	nbi	Control	IC8	0,01149624	0,69	0,0115	*	Wilcoxon
**04.05.2021**	nbi	Control	IC9	0,03546299	1	0,03546	*	Wilcoxon
**04.05.2021**	nbi	IC6	IC7	0,63052891	1	0,63053	ns	Wilcoxon
**04.05.2021**	nbi	IC6	IC8	0,24745069	1	0,24745	ns	Wilcoxon
**04.05.2021**	nbi	IC6	IC9	0,43587218	1	0,43587	ns	Wilcoxon

**04.05.2021**	nbi	IC7	IC8	0,35268137	1	0,35268	ns	Wilcoxon
**04.05.2021**	nbi	IC7	IC9	0,43587218	1	0,43587	ns	Wilcoxon
**04.05.2021**	nbi	IC8	IC9	0,27986101	1	0,27986	ns	Wilcoxon
**11.05.2021**	nbi	Control	IC6	0,24745069	1	0,24745	ns	Wilcoxon
**11.05.2021**	nbi	Control	IC7	0,00287947	0,17	0,00288	**	Wilcoxon
**11.05.2021**	nbi	Control	IC8	0,02323064	1	0,02323	*	Wilcoxon
**11.05.2021**	nbi	Control	IC9	0,00032475	0,02	0,00032	***	Wilcoxon
**11.05.2021**	nbi	IC6	IC7	0,00388621	0,23	0,00389	**	Wilcoxon
**11.05.2021**	nbi	IC6	IC8	0,02880556	1	0,02881	*	Wilcoxon
**11.05.2021**	nbi	IC6	IC9	1,08E-05	0	1,10E-05	****	Wilcoxon
**11.05.2021**	nbi	IC7	IC8	0,12300548	1	0,12301	ns	Wilcoxon
**11.05.2021**	nbi	IC7	IC9	0,24745069	1	0,24745	ns	Wilcoxon
**11.05.2021**	nbi	IC8	IC9	0,00105003	0,06	0,00105	**	Wilcoxon
**18.05.2021**	nbi	Control	IC6	0,39304813	1	0,39305	ns	Wilcoxon
**18.05.2021**	nbi	Control	IC7	0,00105003	0,06	0,00105	**	Wilcoxon
**18.05.2021**	nbi	Control	IC8	0,00208924	0,13	0,00209	**	Wilcoxon
**18.05.2021**	nbi	Control	IC9	4,33E-05	0	4,30E-05	****	Wilcoxon
**18.05.2021**	nbi	IC6	IC7	0,00388621	0,23	0,00389	**	Wilcoxon
**18.05.2021**	nbi	IC6	IC8	0,00684146	0,41	0,00684	**	Wilcoxon
**18.05.2021**	nbi	IC6	IC9	0,0001299	0,01	0,00013	***	Wilcoxon
**18.05.2021**	nbi	IC7	IC8	0,21756262	1	0,21756	ns	Wilcoxon
**18.05.2021**	nbi	IC7	IC9	0,04325705	1	0,04326	*	Wilcoxon
**18.05.2021**	nbi	IC8	IC9	0,02880556	1	0,02881	*	Wilcoxon
**25.05.2021**	nbi	Control	IC6	0,12300548	1	0,12301	ns	Wilcoxon
**25.05.2021**	nbi	Control	IC7	0,00287947	0,17	0,00288	**	Wilcoxon
**25.05.2021**	nbi	Control	IC8	0,57874169	1	0,57874	ns	Wilcoxon
**25.05.2021**	nbi	Control	IC9	0,00048713	0,03	0,00049	***	Wilcoxon
**25.05.2021**	nbi	IC6	IC7	0,00032475	0,02	0,00032	***	Wilcoxon
**25.05.2021**	nbi	IC6	IC8	0,06301284	1	0,06301	ns	Wilcoxon
**25.05.2021**	nbi	IC6	IC9	7,58E-05	0	7,60E-05	****	Wilcoxon
**25.05.2021**	nbi	IC7	IC8	0,06301284	1	0,06301	ns	Wilcoxon
**25.05.2021**	nbi	IC7	IC9	0,22630143	1	0,2263	ns	Wilcoxon
**25.05.2021**	nbi	IC8	IC9	0,02880556	1	0,02881	*	Wilcoxon
**08.06.2021**	nbi	Control	IC6	0,63052891	1	0,63053	ns	Wilcoxon
**08.06.2021**	nbi	Control	IC7	0,00514218	0,31	0,00514	**	Wilcoxon
**08.06.2021**	nbi	Control	IC8	0,00105003	0,06	0,00105	**	Wilcoxon
**08.06.2021**	nbi	Control	IC9	0,01468964	0,88	0,01469	*	Wilcoxon
**08.06.2021**	nbi	IC6	IC7	0,02880556	1	0,02881	*	Wilcoxon
**08.06.2021**	nbi	IC6	IC8	0,01468964	0,88	0,01469	*	Wilcoxon
**08.06.2021**	nbi	IC6	IC9	0,0524259	1	0,05243	ns	Wilcoxon
**08.06.2021**	nbi	IC7	IC8	0,52884886	1	0,52885	ns	Wilcoxon
**08.06.2021**	nbi	IC7	IC9	0,35268137	1	0,35268	ns	Wilcoxon
**08.06.2021**	nbi	IC8	IC9	0,12300548	1	0,12301	ns	Wilcoxon
**22.06.2021**	nbi	Control	IC6	0,00462773	0,28	0,00463	**	Wilcoxon
**22.06.2021**	nbi	Control	IC7	0,0089307	0,54	0,00893	**	Wilcoxon
**22.06.2021**	nbi	Control	IC8	0,19031588	1	0,19032	ns	Wilcoxon
**22.06.2021**	nbi	Control	IC9	0,36384204	1	0,36384	ns	Wilcoxon
**22.06.2021**	nbi	IC6	IC7	0,16125051	1	0,16125	ns	Wilcoxon

**22.06.2021**	nbi	IC6	IC8	0,07023858	1	0,07024	ns	Wilcoxon
**22.06.2021**	nbi	IC6	IC9	0,00699301	0,42	0,00699	**	Wilcoxon
**22.06.2021**	nbi	IC7	IC8	0,39304813	1	0,39305	ns	Wilcoxon
**22.06.2021**	nbi	IC7	IC9	0,0185109	1	0,01851	*	Wilcoxon
**22.06.2021**	nbi	IC8	IC9	0,08782394	1	0,08782	ns	Wilcoxon

Supplementary Table S3. Pairwise PERMANOVA shows if the influence of treatments on the bacterial community composition is significantly different between the respective treatments. Formula = pairwise.adonis2(bray ~ Treatment, data = braydf, p.adjust.m = "bonferroni"), with Bonferroni correction to adjust p-values for multiple testing. Treatments are the uninoculated control and inoculated treatments from $2.5*10^6$ CFU/mL to $2.5*10^9$ CFU/mL (IC6 - IC9). n = 5.

IC6_vs_IC7

	Df	SumOfSqs	R2	F	Pr(>F)
Treatment	1	0.20274	0.28732	3.2253	0.01 **
Residual	8	0.50288	0.71268		
Total	9	0.70562	1.00000		

IC6_vs_IC8

	Df	SumOfSqs	R2	F	Pr(>F)
Treatment	1	0.18415	0.22677	2.3463	0.038 *
Residual	8	0.62788	0.77323		
Total	9	0.81203	1.00000		

IC6_vs_IC9

	Df	SumOfSqs	R2	F	Pr(>F)
Treatment	1	0.16282	0.21258	2.4297	0.004 **
Residual	9	0.60311	0.78742		
Total	10	0.76594	1.00000		

IC6_vs_control

	Df	SumOfSqs	R2	F	Pr(>F)
Treatment	1	0.23301	0.24745	2.6305	0.01 **
Residual	8	0.70863	0.75255		
Total	9	0.94165	1.00000		

IC7_vs_IC8

	Df	SumOfSqs	R2	F	Pr(>F)
Treatment	1	0.06929	0.106	0.9486	0.419
Residual	8	0.58442	0.894		
Total	9	0.65371	1.000		

IC7_vs_IC9

	Df	SumOfSqs	R2	F	Pr(>F)
Treatment	1	0.13761	0.19736	2.213	0.019 *
Residual	9	0.55965	0.80264		
Total	10	0.69727	1.00000		

IC7_vs_control

	Df	SumOfSqs	R2	F	Pr(>F)
Treatment	1	0.15705	0.19101	1.8888	0.01 **
Residual	8	0.66517	0.80899		
Total	9	0.82222	1.00000		

IC8_vs_IC9

	Df	SumOfSqs	R2	F	Pr(>F)
Treatment	1	0.08586	0.11143	1.1286	0.287
Residual	9	0.68466	0.88857		
Total	10	0.77052	1.00000		

IC8_vs_control

	Df	SumOfSqs	R2	F	Pr(>F)
Treatment	1	0.16599	0.1736	1.6805	0.066 .
Residual	8	0.79018	0.8264		
Total	9	0.95617	1.0000		

IC9_vs_control

	Df	SumOfSqs	R2	F	Pr(>F)
Treatment	1	0.23997	0.23868	2.8216	0.004 **
Residual	9	0.76541	0.76132		
Total	10	1.00538	1.00000		

Signif. codes: 0 ‘***’ 0.001 ‘**’ 0.01 ‘*’ 0.05 ‘.’ 0.1 ‘ ’ 1

Supplementary Table S4. Pairwise PERMANOVA of only inoculated samples shows if the influence of treatments on the bacterial community composition is significantly different between the respective treatments. Formula = pairwise.adonis2(bray ~ Treatment, data = braydf, p.adjust.m = "bonferroni"), with Bonferroni correction to adjust p-values for multiple testing. Treatments are inoculated treatments from $2.5*10^{6}$ CFU/mL to $2.5*10^{9}$ CFU/mL (IC6 - IC9). n = 5.

IC6_vs_IC7

	Df	SumOfSqs	R2	F	Pr(>F)
Treatment	1	0.20274	0.28732	3.2253	0.012 *
Residual	8	0.50288	0.71268		
Total	9	0.70562	1.00000		

IC6_vs_IC8

	Df	SumOfSqs	R2	F	Pr(>F)
Treatment	1	0.18415	0.22677	2.3463	0.032 *
Residual	8	0.62788	0.77323		
Total	9	0.81203	1.00000		

IC6_vs_IC9

	Df	SumOfSqs	R2	F	Pr(>F)
Treatment	1	0.16282	0.21258	2.4297	0.005 **
Residual	9	0.60311	0.78742		
Total	10	0.76594	1.00000		

IC7_vs_IC8

	Df	SumOfSqs	R2	F	Pr(>F)
Treatment	1	0.06929	0.106	0.9486	0.442
Residual	8	0.58442	0.894		
Total	9	0.65371	1.000		

IC7_vs_IC9

	Df	SumOfSqs	R2	F	Pr(>F)
Treatment	1	0.13761	0.19736	2.213	0.021 *
Residual	9	0.55965	0.80264		
Total	10	0.69727	1.00000		

IC8_vs_IC9

	Df	SumOfSqs	R2	F	Pr(>F)
Treatment	1	0.08586	0.11143	1.1286	0.307
Residual	9	0.68466	0.88857		
Total	10	0.77052	1.00000		

Signif. codes: 0 ‘***’ 0.001 ‘**’ 0.01 ‘*’ 0.05 ‘.’ 0.1 ‘ ’ 1

Supplementary Table S5. P-Values of significant different relative abundances for the top 30 genera using a pairwise Wilcoxon test. After correction for multiple testing using Bonferroni correction, only *Rhodanobacter* was significantly different for Control vs. IC9 and IC7 vs. IC9.

GENUS	GROUP1	GROUP2	P	P.ADJ	P.ADJ.SIGNIF
ACIDOTHERMUS	IC6	IC7	0,008	0,079	ns
ACIDOTHERMUS	IC6	IC8	0,032	0,317	ns
ARTHROBACTER	IC6	IC8	0,016	0,159	ns
ARTHROBACTER	IC8	IC9	0,03	0,303	ns
BRADYRHIZOBIUM	Control	IC6	0,008	0,079	ns
BRADYRHIZOBIUM	IC6	IC7	0,008	0,079	ns
BRADYRHIZOBIUM	IC7	IC8	0,016	0,159	ns
BRADYRHIZOBIUM	IC7	IC9	0,03	0,303	ns
BURKHOLDERIA-CABALLERONIA-PARABURKHOLDERIA	Control	IC9	0,009	0,087	ns
BURKHOLDERIA-CABALLERONIA-PARABURKHOLDERIA	IC7	IC9	0,017	0,173	ns
BURKHOLDERIA-CABALLERONIA-PARABURKHOLDERIA	IC8	IC9	0,009	0,087	ns
CANDIDATUS UDAEOBACTER	Control	IC9	0,009	0,087	ns
GEMMATIMONAS	Control	IC9	0,009	0,087	ns
GEMMATIMONAS	Control	IC6	0,016	0,159	ns
GEMMATIMONAS	Control	IC7	0,032	0,317	ns
RHODANOBACTER	Control	IC9	0,004	0,043	*
RHODANOBACTER	IC7	IC9	0,004	0,043	*
RHODANOBACTER	Control	IC6	0,008	0,079	ns
RHODANOBACTER	Control	IC8	0,032	0,317	ns
RHODANOBACTER	IC6	IC7	0,008	0,079	ns
STREPTOMYCES	IC6	IC7	0,008	0,079	ns
STREPTOMYCES	IC6	IC8	0,008	0,079	ns
TERRABACTER	IC6	IC8	0,008	0,079	ns
**U. ACIDOBACTERIALES**	IC6	IC9	0,017	0,173	ns
**U. ACIDOBACTERIALES**	IC7	IC9	0,03	0,303	ns
**U. CHLOROFLEXI**	Control	IC6	0,008	0,079	ns
**U. CHLOROFLEXI**	Control	IC9	0,009	0,087	ns
**U. CHLOROFLEXI**	Control	IC8	0,032	0,317	ns
**U. GAIELLALES**	IC6	IC7	0,032	0,317	ns
**U. GEMMATIMONADACEAE**	IC7	IC9	0,009	0,087	ns
**U. LWQ8**	Control	IC7	0,032	0,317	ns
**U. LWQ8**	IC7	IC9	0,03	0,303	ns
**U. VICINAMIBACTERALES**	Control	IC9	0,03	0,303	ns
**U. VICINAMIBACTERALES**	IC7	IC9	0,017	0,173	ns
**U. WD2101 SOIL GROUP**	Control	IC6	0,008	0,079	ns
**U. WD2101 SOIL GROUP**	Control	IC9	0,03	0,303	ns
**U. WD2101 SOIL GROUP**	IC6	IC7	0,008	0,079	ns
**U. WD2101 SOIL GROUP**	IC7	IC9	0,03	0,303	ns

Supplementary Table S6. Hubs of bacterial co-occurrence network IC9, calculated using Spearman correlations and the NetCoMi R-package. We used centered log-ratio (clr) transformation for data normalization, handled zeros by pseudo count, and set a 0.3 correlation coefficient threshold as sparsification method.

HUBS IN IC9 NETWORK	PHYLUM	CLASS	ORDER	FAMILY	GENUS
ASV1989	Acidobacteriota	Acidobacteriae	Acidobacteriales	U. Acidobacteriales	U. Acidobacteriales
ASV3491	Acidobacteriota	Acidobacteriae	Solibacterales	Solibacteraceae	Candidatus Solibacter
ASV3505	Acidobacteriota	Acidobacteriae	Bryobacterales	Bryobacteraceae	Bryobacter
ASV3571	Actinobacteriota	Thermoleophilia	Gaiellales	U. Gaiellales	U. Gaiellales
ASV3581	Actinobacteriota	Thermoleophilia	Solirubrobacterales	67-14	U. 67-14
ASV3582	Actinobacteriota	Thermoleophilia	Solirubrobacterales	67-14	U. 67-14
ASV3687	Actinobacteriota	Actinobacteria	Streptosporangiales	Streptosporangiaceae	Streptosporangium
ASV3704	Actinobacteriota	Actinobacteria	Streptosporangiales	Streptosporangiaceae	Microbispora
ASV4026	Actinobacteriota	Thermoleophilia	Gaiellales	U. Gaiellales	U. Gaiellales
ASV4056	Actinobacteriota	Thermoleophilia	Gaiellales	U. Gaiellales	U. Gaiellales
ASV4062	Actinobacteriota	Thermoleophilia	Gaiellales	U. Gaiellales	U. Gaiellales
ASV4140	Actinobacteriota	Thermoleophilia	Gaiellales	Gaiellaceae	Gaiella
ASV4224	Armatimonadota	Armatimonadia	Armatimonadales	U. Armatimonadales	U. Armatimonadales
ASV3271	Chloroflexi	Ktedonobacteria	B12-WMSP1	U. B12-WMSP1	U. B12-WMSP1
ASV3294	Chloroflexi	Ktedonobacteria	Ktedonobacterales	JG30-KF-AS9	U. JG30-KF-AS9
ASV3307	Chloroflexi	Ktedonobacteria	Ktedonobacterales	JG30-KF-AS9	U. JG30-KF-AS9
ASV4449	Chloroflexi	Ktedonobacteria	Ktedonobacterales	Ktedonobacteraceae	HSB OF53-F07
ASV4690	Chloroflexi	Chloroflexia	Thermomicrobiales	Thermomicrobiaceae	Nitrolancea
ASV5348	Chloroflexi	KD4-96	U. KD4-96	U. KD4-96	U. KD4-96
ASV5415	Chloroflexi	JG30-KF-CM66	U. JG30-KF-CM66	U. JG30-KF-CM66	U. JG30-KF-CM66
ASV5523	Chloroflexi	KD4-96	U. KD4-96	U. KD4-96	U. KD4-96
ASV4132	Firmicutes	Bacilli	Paenibacillales	Paenibacillaceae	Ammoniphilus
ASV4359	Firmicutes	Limnochordia	Hydrogenispora	U. Hydrogenispora	U. Hydrogenispora
ASV4361	Firmicutes	Symbiobacteriia	Symbiobacteriales	Symbiobacteraceae	Symbiobacterium
ASV4399	Firmicutes	Bacilli	Bacillales	Bacillaceae	Geobacillus
ASV5207	Firmicutes	Bacilli	Bacillales	Bacillaceae	Bacillus
ASV5219	Firmicutes	Bacilli	Bacillales	Bacillaceae	Bacillus
ASV5269	Firmicutes	Bacilli	Bacillales	Bacillaceae	Bacillus
ASV5275	Firmicutes	Bacilli	Bacillales	Bacillaceae	Bacillus
ASV5279	Firmicutes	Bacilli	Bacillales	Planococcaceae	Lysinibacillus
ASV5298	Firmicutes	Bacilli	Bacillales	Bacillaceae	Bacillus
ASV5452	Firmicutes	Clostridia	Oscillospirales	Hungateiclostridiaceae	UCG-012
ASV3119	Halanaerobiaeota	Halanaerobiia	Halanaerobiales	Halanaerobiaceae	Halocella
ASV564	Myxococcota	bacteriap25	U. bacteriap25	U. bacteriap25	U. bacteriap25
ASV2189	Patescibacteria	Saccharimonadia	Saccharimonadales	LWQ8	U. LWQ8
ASV1512	Proteobacteria	Alphaproteobacteria	Rhizobiales	Xanthobacteraceae	Pseudolabrys
ASV2395	Proteobacteria	Alphaproteobacteria	Sphingomonadales	Sphingomonadaceae	Sphingomonas

Supplementary Figures

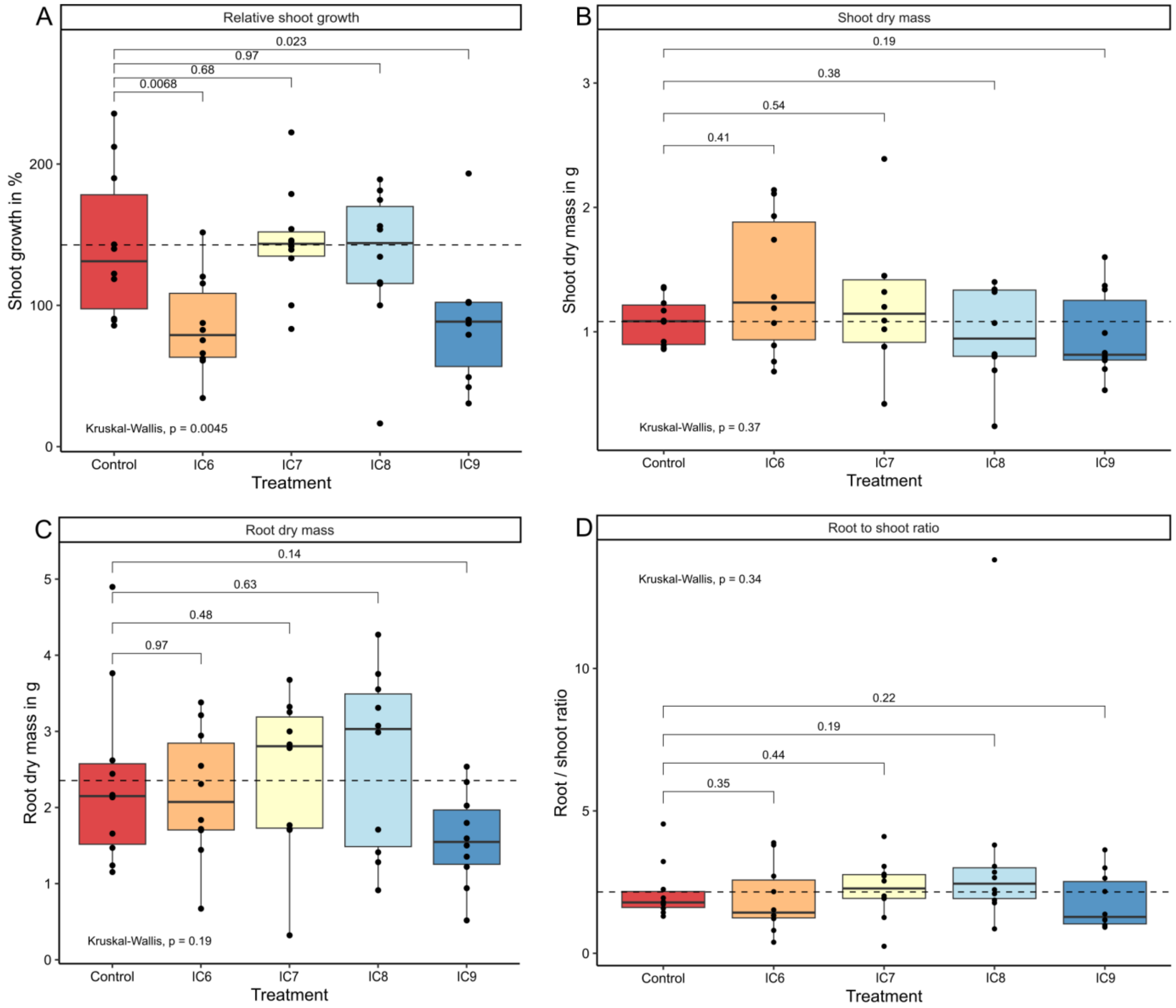

Supplementary Figure S1. Apple plant growth parameter after 8 weeks growth in ARD soil in the greenhouse. Treatments are the uninoculated control and inoculated treatments from $2.5*10^6$ CFU/mL to $2.5*10^9$ CFU/mL (IC6 - IC9). n = 10. The significance was tested using Kruskal-Wallis and Wilcoxon Rank-Sum test with Bonferroni correction for multiple testing. A) shoot growth in [%], B) shoot dry mass in [g], C) root dry mass in [g], D) root to shoot ratio

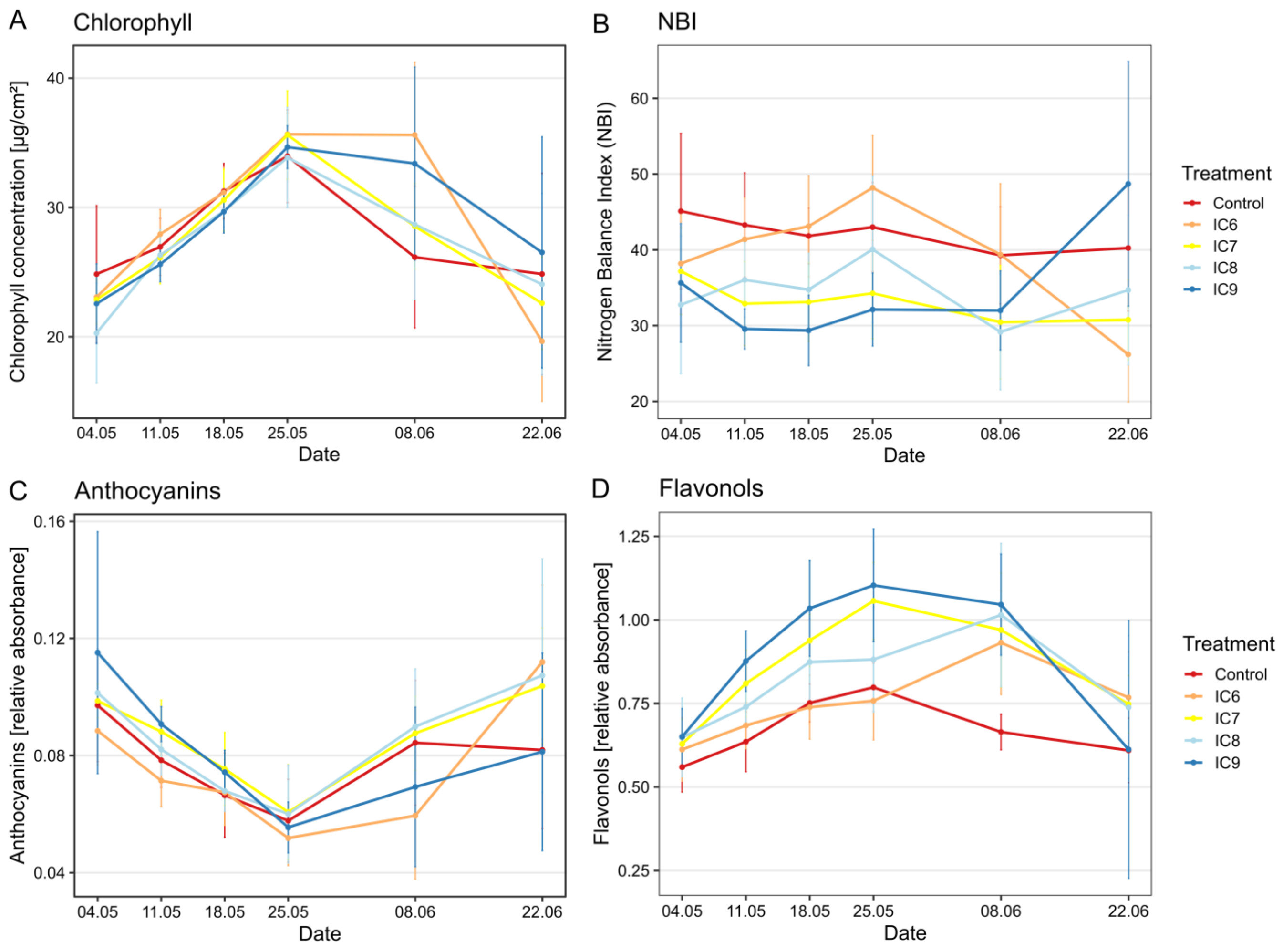

Supplementary Figure S2. Fluorescence measurements of chlorophyll, flavonols, anthocyanins and nitrogen balance index (NBI) in young apple plantlet leaves during 8 weeks growth in ARD soil in the greenhouse. Treatments are the uninoculated control and inoculated treatments from $2.5*10^6$ CFU/mL to $2.5*10^9$ CFU/mL (IC6 - IC9). Shown are mean values (points) of n = 10 replicates, with standard deviation. A) chlorophyll concentration in [µg/cm^2], B) NBI (ratio of chlorophyll to flavonol content, C) anthocyanins [relative absorbance], D) flavonols [relative absorbance]

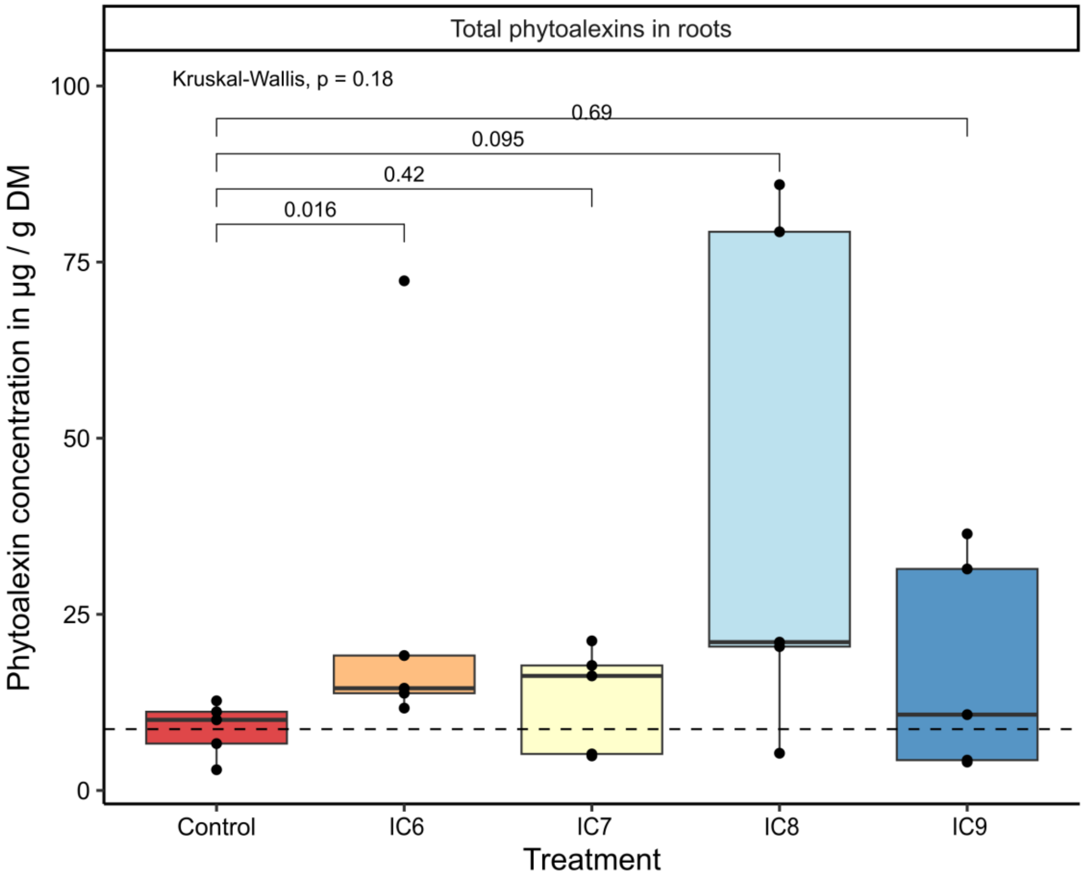

Supplementary Figure S3. Total phytoalexin concentration of apple roots grown for 8 weeks in ARD soil in the greenhouse. Treatments are the uninoculated control and inoculated treatments from $2.5*10^6$ CFU/mL to $2.5*10^9$ CFU/mL (IC6 - IC9). n = 5. The significance was tested using Kruskal-Wallis and Wilcoxon Rank-Sum test with Bonferroni correction for multiple testing. DM = dry mass.

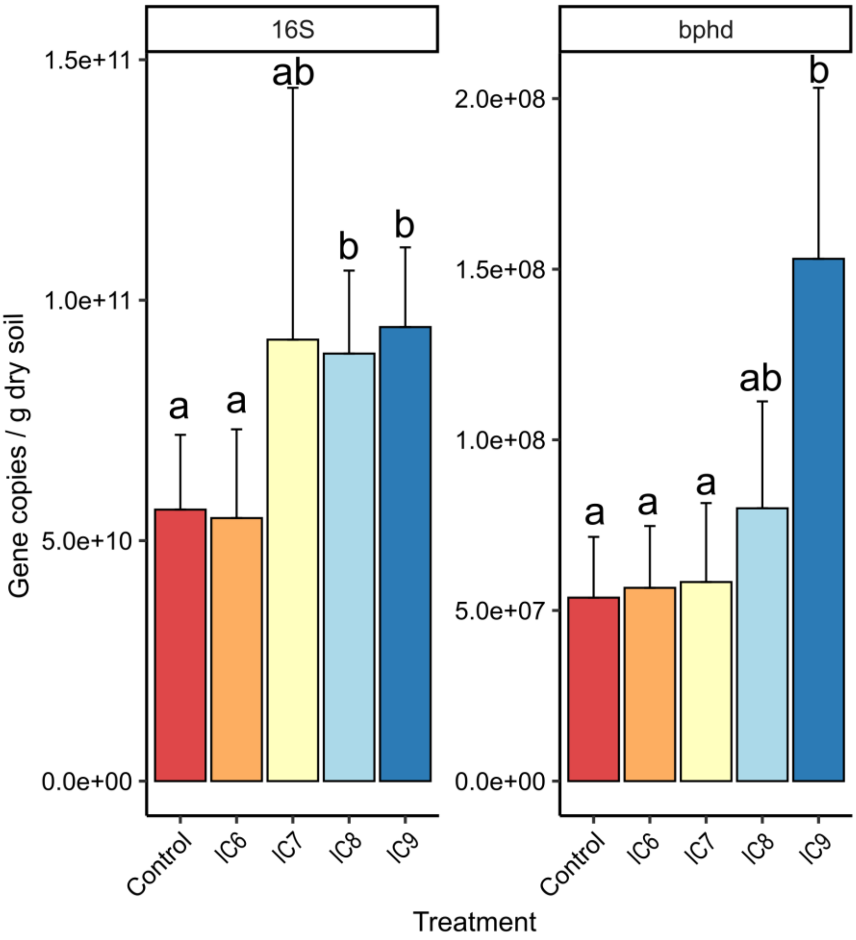

Supplementary Figure S4. Absolute numbers of gene copies per g soil for the partial 16S rRNA gene and biphenyl dioxygenases (*bphd*) in the bacterial rhizosphere communities for control (uninoculated samples) and inoculated samples using different concentrations of *R. pseudokoreensis* R79^T (10^6 – 10^9 CFU/mL = IC6 – IC9). qPCR was done using total community DNA. For 16S rRNA gene qPCR, control was significantly different to IC8 (P = 0.032) and IC9 (p = 0.016); for *bphd* qPCR, control was significantly different to IC9 (p = 0.0159).

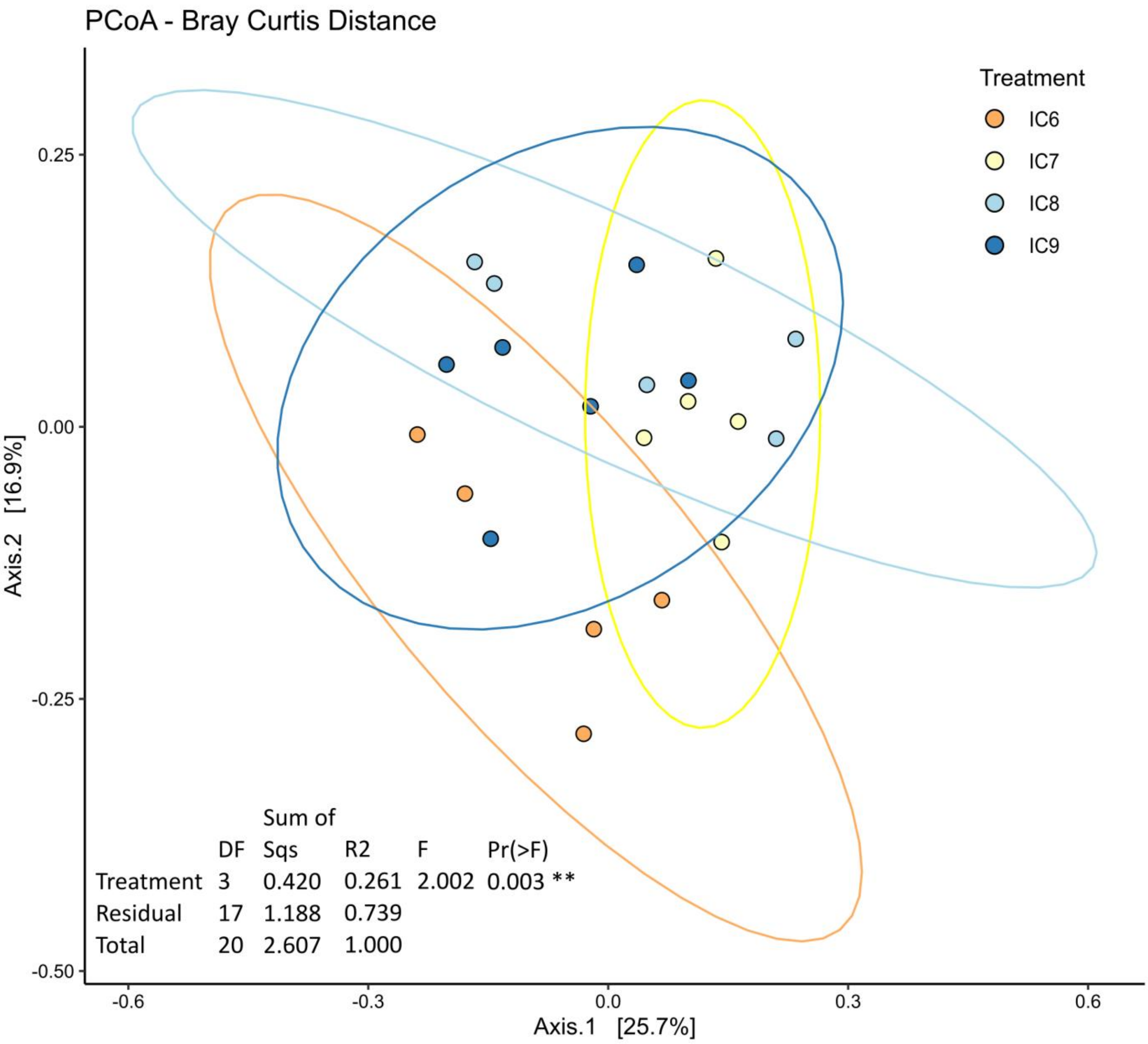

Supplementary Figure S5. Bacterial community structure: PCoA plot of Bray-Curtis distances showing the beta diversity of only inoculated treatments from 2.5*10^6 CFU/mL to 2.5*10^9 CFU/mL (IC6 - IC9). Statistical analysis was performed using PERMANOVA with Bonferroni correction for multiple testing. Differences were considered as significant with $p<0.05$.

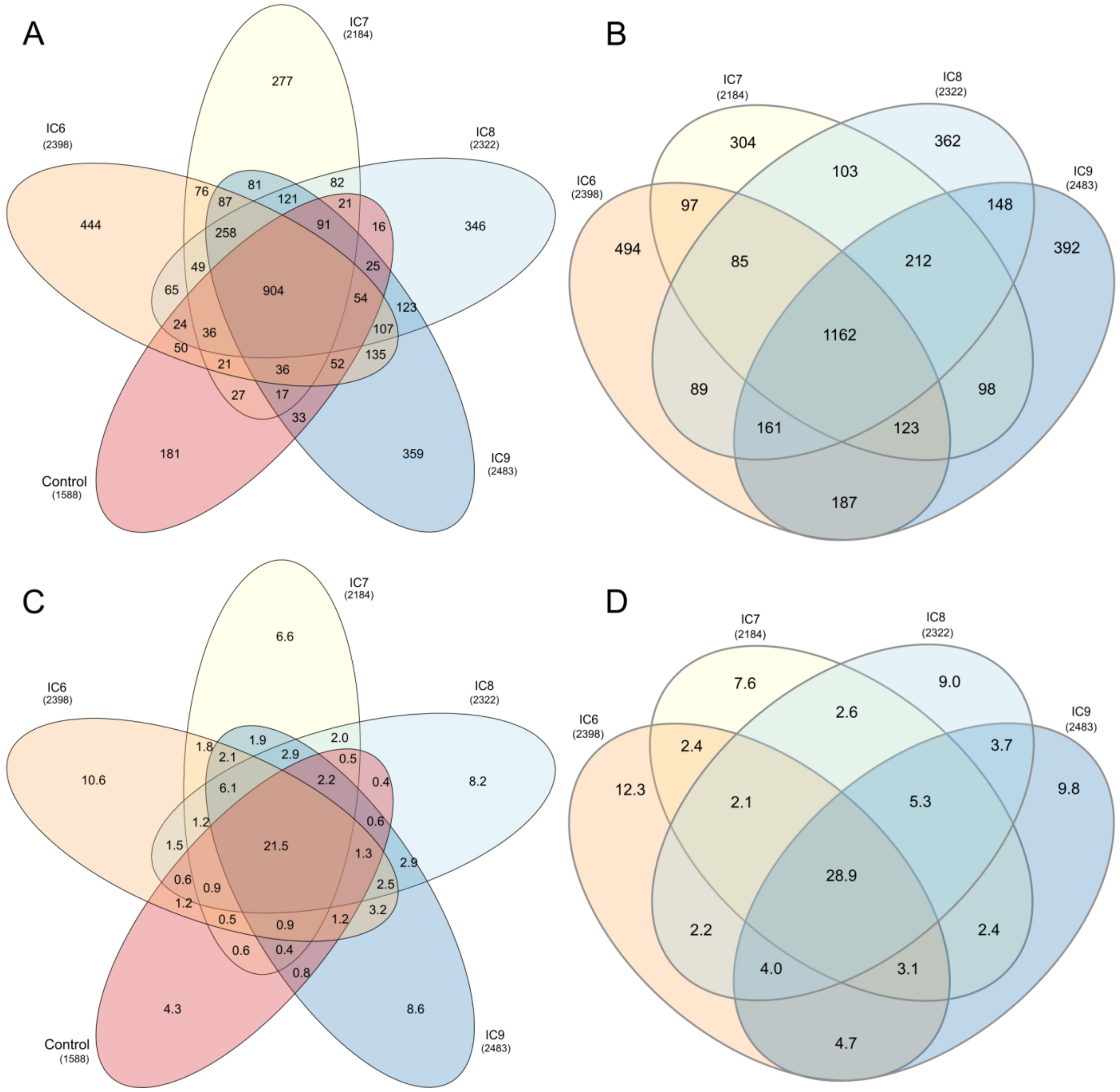

Supplementary Figure S6. Venn Diagrams of shared ASVs of the treatments of the uninoculated control and inoculated treatments from 2.5*10^6 CFU/mL to 2.5*10^9 CFU/mL (IC6 - IC9). ASVs had to be in 4/5 replicates to be considered. Venn diagrams were made using InteractiVenn (5). A: All treatments in total numbers, B: Only inoculated treatments in total numbers, C: All treatments in percent, D: Only inoculated treatments in percent.

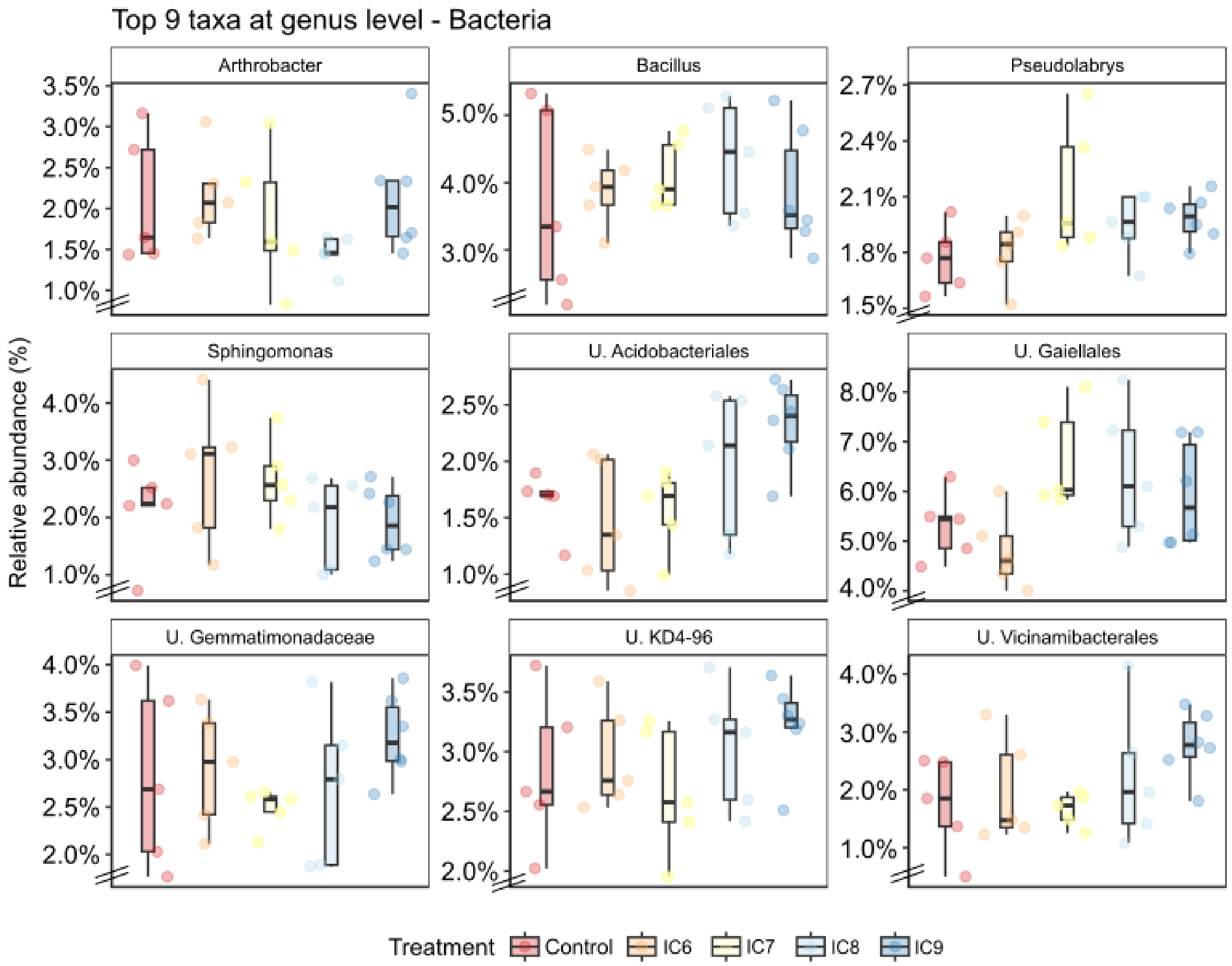

Supplementary Figure S7. Relative abundances of the top 9 genera in uninoculated control and treatments with inoculation of *Rhodococcus pseudokoreensis* R79^T at concentrations from 2.5*10^6 CFU/mL to 2.5*10^9 CFU/mL (IC6 - IC9). U. indicates unknown member (family) of this (higher) taxonomic group. Names consisting of numbers and letters are taxa belonging to groups without validly published scientific names, mostly coming from sequencing data. The graph was constructed using the microbiomeutilities v 1.00.17 R-package.

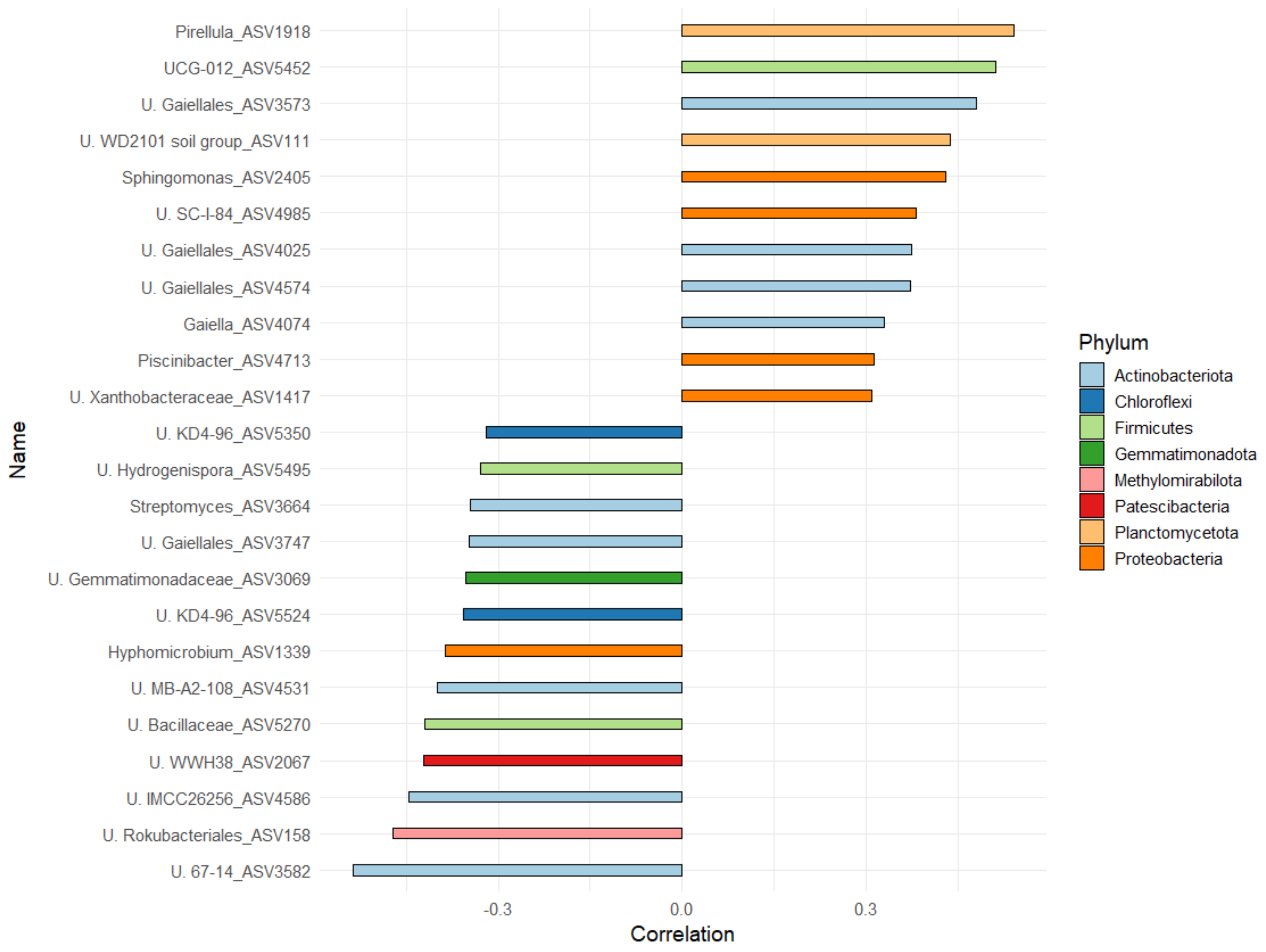

Supplementary Figure S8. ASVs significantly correlated to inoculation load (concentration of inoculum in CFU/mL, not-inoculated control was implemented with a load = 0). ASVs were filtered beforehand to contain only ASVs present in all treatments. Displayed are only taxa with a correlation value > |0.3|. Labels are ASVs with their Genus-level name, coloured by Phylum. Unknown taxonomic level is always described as unknown (U.) member of the next higher known taxonomic level.

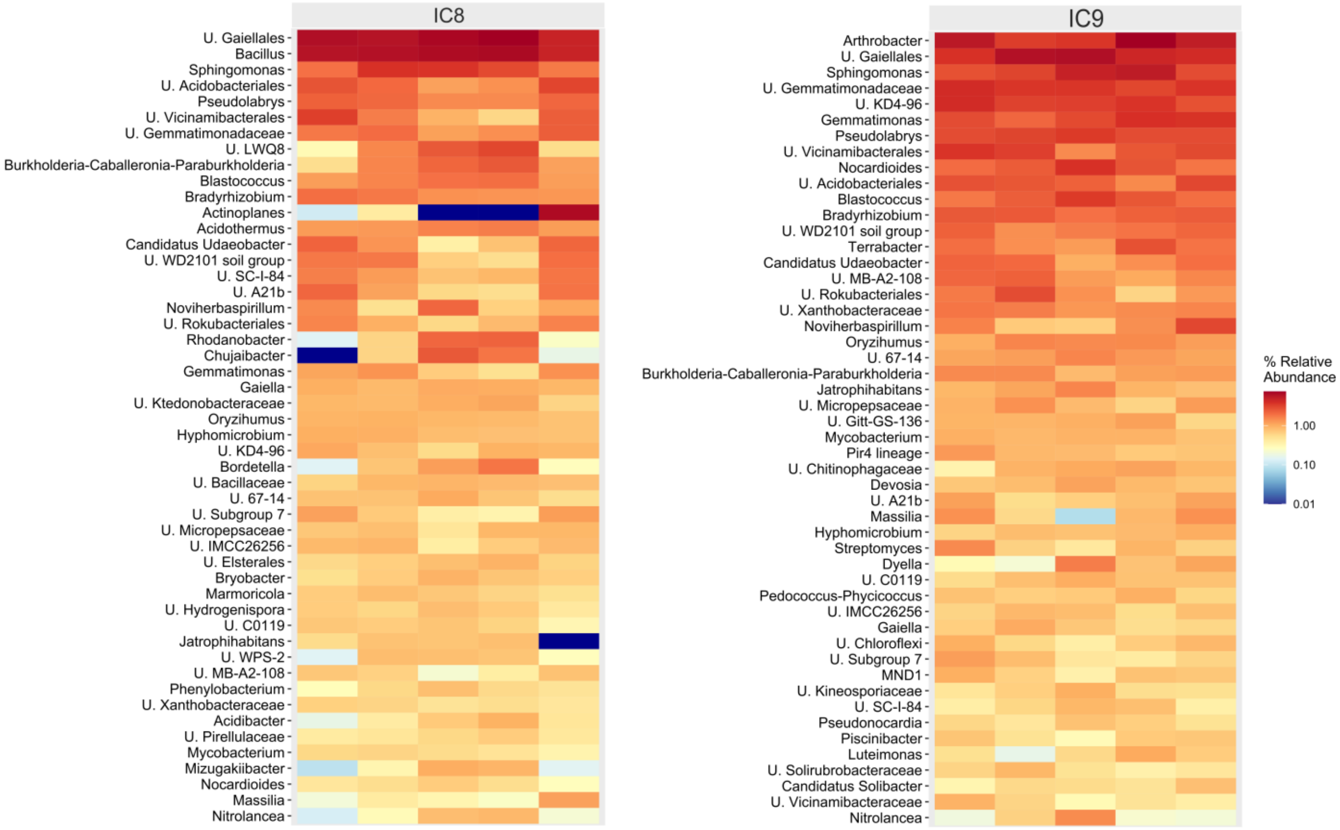

Supplementary Figure S9. Taxonomy of nodes connected to *R. pseudokoreensis* R79^{T} in the bacterial co-occurrence networks of IC8 and IC9 aggregated on genus level. Shown are only the top 50 genera. Columns are individual replicates.

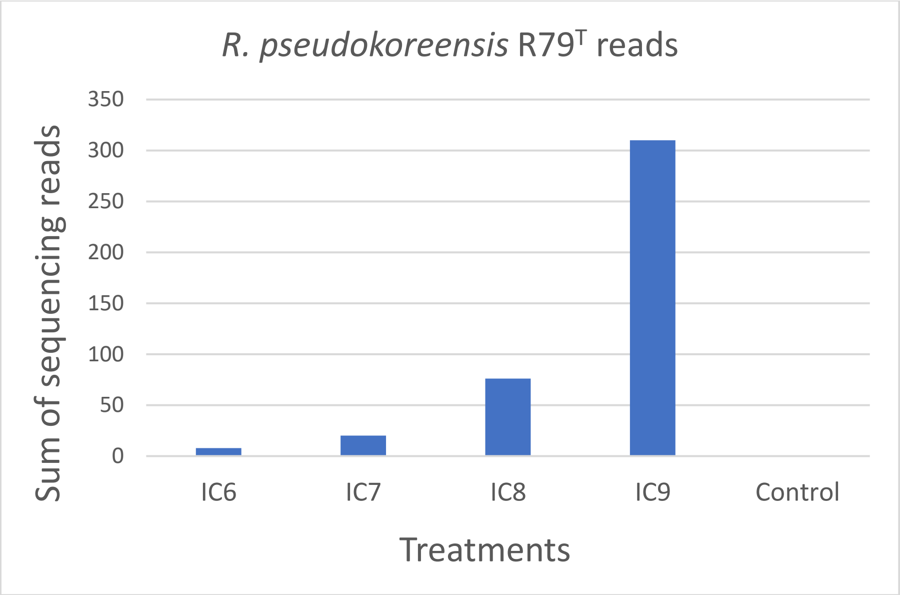

Supplementary Figure S10. Sum of sequencing reads per treatment assigned to inoculant *R. pseudokoreensis* R79^{T}, ASV4809

Supplementary References

1. Parada AE, Needham DM, Fuhrman JA. Every base matters: Assessing small subunit rRNA primers for marine microbiomes with mock communities, time series and global field samples. Environ Microbiol. 2016;18:1403–14.

2. Apprill A, Mcnally S, Parsons R, Weber L. Minor revision to V4 region SSU rRNA 806R gene primer greatly increases detection of SAR11 bacterioplankton. Aquatic Microbial Ecology 2015;75:129–37.

3. Bach HJ, Tomanova J, Schloter M, Munch JC. Enumeration of total bacteria and bacteria with genes for proteolytic activity in pure cultures and in environmental samples by quantitative PCR mediated amplification. J Microbiol Methods. 2002;49:235–45.

4. Iwai S, Chai B, Sul WJ, Cole JR, Hashsham SA, Tiedje JM. Gene-targeted-metagenomics reveals extensive diversity of aromatic dioxygenase genes in the environment. ISME Journal. 2010;4:279–85.

5. Heberle H, Meirelles VG, da Silva FR, Telles GP, Minghim R. InteractiVenn: A web-based tool for the analysis of sets through Venn diagrams. BMC Bioinformatics. 2015;16:1–7.
